# SVEP1 enables efficient binding of angiopoietin-2 to the TIE1 receptor, allowing receptor phosphorylation and downstream signaling

**DOI:** 10.7554/eLife.106338

**Published:** 2026-10-30

**Authors:** Katharina Uphoff, Melina Hußmann, Oliver Peters, Matthias Mörgelin, Fabian Metzen, Wolfgang MJ Obermann, Fernando J Bazan, Jörg Stetefeld, Manuel Koch, Stefan Schulte-Merker, Dörte Schulte-Ostermann

**Affiliations:** 1 Institute for Cardiovascular Organogenesis and Regeneration, Faculty of Medicine, University of Münster Münster Germany; 2 Department of Chemistry, University of Manitoba Winnipeg Canada; 3 Colzyx AB Lund Sweden; 4 Institute for Dental Research and Oral Musculoskeletal Biology, Faculty of Medicine and University Hospital Cologne, University of Cologne Cologne Germany; 5 ħ bioconsulting llc Stillwater United States; Goethe University Frankfurt Germany; Goethe University Frankfurt Germany

**Keywords:** vascular biology, lymphangiogenesis, growth factors, receptors, signaling, cell biology, Human

## Abstract

The molecular mechanisms that drive (lymph-)angiogenesis are crucial to understanding diseases, such as lymphedema, that are caused due to malformations of the lymphatic vasculature. Recently, an interaction between the secreted protein Svep1, a key regulator in lymphangiogenesis, and the transmembrane receptor Tie1 was shown in zebrafish, humans, and mice. Here, guided by in silico AlphaFold-multimer structure predictions of SVEP1 complexes, we assert with protein binding studies that the human CCP20 domain is the primary binding site for TIE1. We further demonstrate that SVEP1 mediates strong binding of ANG2 and TIE1, and that combined stimulation of hdLECs with SVEP1 and ANG2 leads to phosphorylation of TIE1. TIE1 activation by SVEP1 and ANG2 enables downstream signaling and, in turn, potentiates nuclear exclusion of FOXO1 and phosphorylation of AKT compared to SVEP1 or ANG2 alone. We present a model in which ANG1/2 dimers bind to both SVEP1 and TIE1, resulting in the recruitment of multiple TIE1 receptor molecules to a multimeric complex at the cell membrane, potentially amplifying its signaling capacity.

## Introduction

Lymphatic vessels are closely associated with blood vessels, and malformations of the lymphatic vasculature lead to lymphedema, obesity, or chronic inflammatory diseases (Mäkinen et al., 2021; Petrova and Koh, 2018; Sunil Kumar et al., 2026). Next to the well-described lymphatic VEGFC/VEGFR3 pathway (Hogan et al., 2009; Bos et al., 2011; Le Guen et al., 2014; Jeltsch et al., 2014; Roukens et al., 2015), it was shown that Sushi, von Willebrand factor type A, EGF, and pentraxin domain containing protein 1 (SVEP1), also referred to as Polydom, is another key player in lymphangiogenesis (Karpanen et al., 2017; Morooka et al., 2017). Svep1 encodes a 3571 amino acid long extracellular matrix protein of different domains like von Willebrand factor type A domain, ephrin-receptor like domains, complex control protein (CCP) domains, and Hyalin repeats at the N-terminus. The C-terminus mainly consists of CCP and EGF domains. Svep1 is expressed in mesenchymal cells and functions non-cell-autonomously (Karpanen et al., 2017; Morooka et al., 2017). Only recently it was discovered that SVEP1 binds to TIE1 (Hußmann et al., 2023; Sato-Nishiuchi et al., 2023), and we were able to provide in vivo evidence for the genetic interaction of *svep1* and *tie1* in zebrafish. Multiple phenotypic hallmarks of *svep1* mutants are phenocopied by *tie1*, but not *tie2* mutants, including (among others) a reduced number of parachordal lymphangioblasts (PLs) (Hußmann et al., 2023). Furthermore, a specific aspect of the facial lymphatics in zebrafish, the facial collecting lymphatic vessel, develops in a *svep1* and *tie1* dependent, but *vegfc/vegfr3* and *tie2*-independent manner (Hußmann et al., 2023).

The discovery of the novel molecular interaction between SVEP1 and TIE1 provided an invitation to further investigate how SVEP1 is linked to the already well-known ANG/TIE pathway. The ANG/TIE pathway triggers a signaling cascade, which is required for lymphatic and blood vessel development, and has a unique role in maintaining vascular stability. Two receptor tyrosine kinases, tyrosine kinase with Ig and EGF domains (TIE1) and tyrosine endothelial kinase (TEK), also known as TIE2 (Dumont et al., 1993; Partanen et al., 1992), are activated through binding of angiopoietin ligands, including ANG1 and ANG2 (Davis et al., 1996; Maisonpierre et al., 1997). TIE1 and TIE2 exhibit a high degree of homology with a globular head domain consisting of three immunoglobulin-like (Ig) domains and three epidermal growth factor-like (EGF) modules and a short stalk formed by three fibronectin type III repeats, while the N-terminal two Ig domains of TIE2 harbor the angiopoietin binding site (Macdonald et al., 2006). TIE2 is considered the main player in angiogenesis, and its phosphorylation, triggered by ANG1 binding, induces downstream signaling via PI3K/Akt, which leads to inhibition of the transcription factor Forkhead box protein O1 (FOXO1) and repression of FOXO1 target genes—such as *Ang2* (Kim et al., 2000; Daly et al., 2004). Although TIE2 is needed for lymphangiogenesis in mouse embryos, with genetic deletions of *Tie2* in lymphatic vessels leading to subcutaneous edema (Souma et al., 2018), postnatal knock-out of *Tie2* in lymphatics of newborn mice only has an effect on lymphatic collecting vessels but not on cutaneous lymphatic capillaries (Korhonen et al., 2022; Shen et al., 2014). While ANG1 is an agonist of TIE2, ANG2 acts as a context-dependent weak agonist or antagonist for TIE2 that can inhibit the ANG1–TIE2 signaling axis (Saharinen et al., 2017). TIE1 blocks the signaling cascade in a context-dependent manner by forming heterodimers with TIE2 (Hansen et al., 2010; Marron et al., 2000; Saharinen et al., 2005; Savant et al., 2015; Seegar et al., 2010). On the other hand, *Tie1* deletion leads to defects in the lymphatic capillary network and primary lymphatic network remodeling (Korhonen et al., 2022; Shen et al., 2014).

In the present study, we extend our understanding of SVEP1 binding to TIE1, utilizing in silico, in vitro, and in vivo techniques. We show that the binding of ANG2 to TIE1 is enhanced by SVEP1, leading to phosphorylation and downstream signaling of TIE1, thereby potentiating phosphorylation of AKT and nuclear exclusion of FOXO1. Our study helps to understand the interaction of important signaling pathway members involved in (lymph-)angiogenesis and provides a model that suggests local TIE1 multimerization through bridging functions of ANG1/2 in the presence of SVEP1.

## Results

### SVEP1 binds to the D1-D2 domain of TIE1

**Figure 1.**
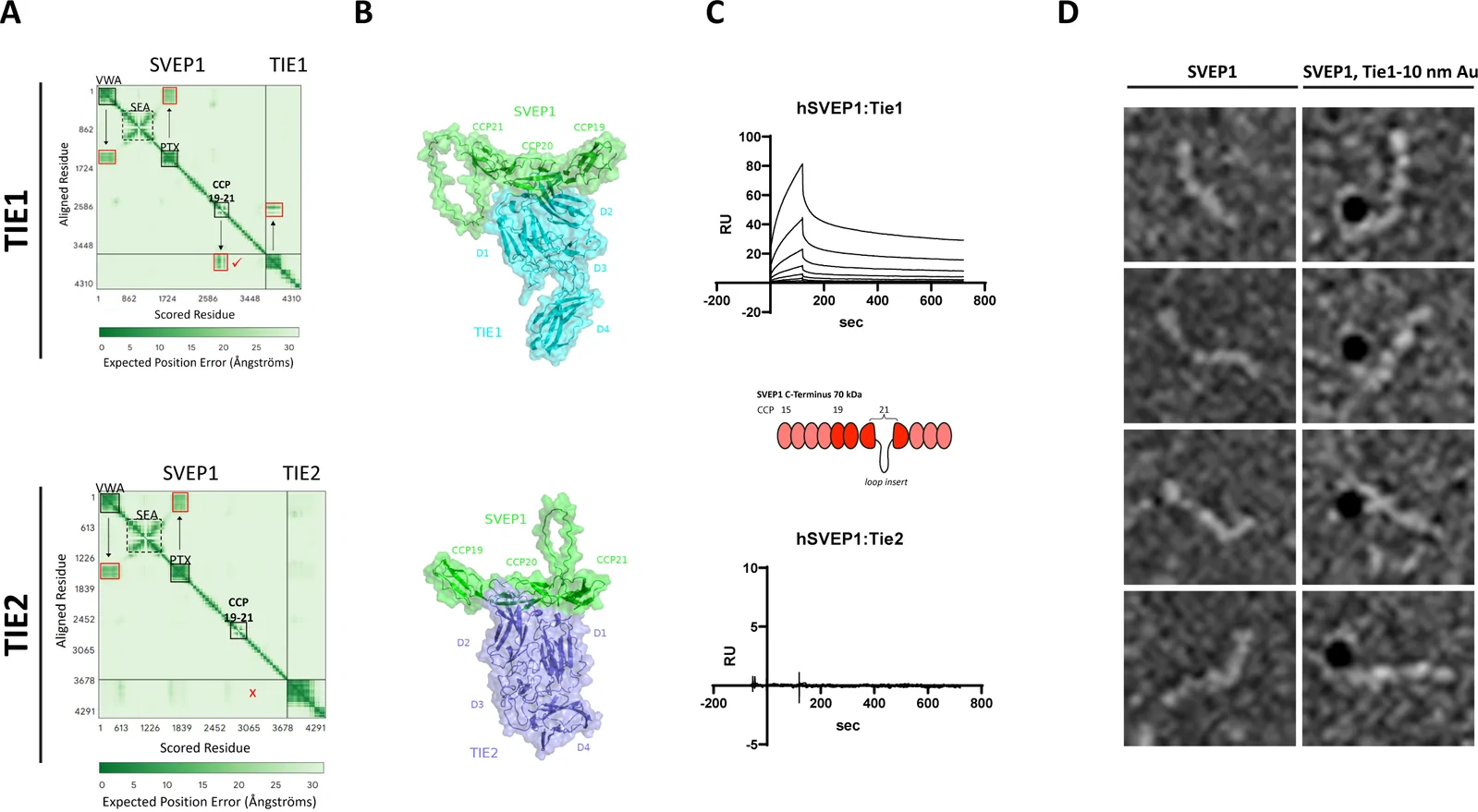
The CCP20 domain of SVEP1 contacts the D1-D2 headpiece domains of TIE1. (**A**) Comparative PAE plots of full-length SVEP1 and the TIE1 ectodomain display a primary binding site for TIE1 (domains D1-D2) by AF3-multimer modeling, that localizes to the CCP19-21 stretch, notably encompassing CCP20. The PAE plot for full-length SVEP1 and the TIE2 ectodomain does not show an equivalent prediction for the same CCP19-21 region. Labeled SVEP1 domains: PTX (Pentraxin), SEA (sperm protein, enterokinase, and agrin) module, VWA (von Willebrand factor type A), statistics flSVEP1-ectoTIE1 pLDDT = 66.1; pTM = 0.34; ipTM = 0.48; statistics flSVEP1-ectoTIE2 pLDDT = 0.68, ipTM = 0.28, pTM = 0.34. (**B**) A discrete AF2.3 model of TIE1 bound to CCP19-21 shows that the Ig-like D1-D2 headpiece domains of TIE1 (linked by a Cys-rich D3 to an ‘open’ or flared-out D4 Ig module) are in contact with CCP20. By contrast, the AF2.3 model of TIE2’s N-terminal domains is more loosely perched between the CCP20 and CCP21 domains with a qualitatively lower affinity due to reduced sidechain contacts. (**C**) Quantitative analysis of TIE1-SVEP1 interaction using SPR assays. In the absence of other proteins, a 70 kD version of SVEP1 (CCP15-CCP24, amino acids: 2261–2890; depicted in diagram fashion) containing the CCP20 domain binds TIE1 (K_D_ = 62.5 nM), but does not bind TIE2. (**D**) Negative staining shows binding of human C-term SVEP1-Strep II (reaching from CCP15 to the C-Term) and TIE1, labeled with 10 nm Au. PAE: predicted aligned error; AF3: AlphaFold 3; CCP: complement control protein (sushi repeats). Figure 1—source data 1.This source data contains PDBs (.cif and .json files) of modelling of the SVEP1/TIE1 complexes shown in Figure 1 and its figure supplements. Figure 1—source data 2.This source data contains PDBs (.cif and .json files) of modelling of the SVEP1/TIE2 complexes shown in Figure 1 and its figure supplements. Figure 1—source data 3.This source data contains PDBs (.cif and .json files) of modelling of the full length SVEP1/TIE1 complexes shown in Figure 1. Figure 1—source data 4.This source data contains PDBs (.cif and .json files) of modelling of the full length SVEP1/TIE1 complexes shown in figure 1.

Previously, it had been shown that solid-phase binding assays of recombinant full-length murine SVEP1 protein display a significant binding of SVEP1 to TIE1, but not TIE2 (Sato-Nishiuchi et al., 2023). In a binary setup, that is, without other proteins being present, we confirmed this observation utilizing surface plasmon resonance (SPR) technology and negative staining assays (Figure 1C and D). Furthermore, and since AlphaFold2 (AF2) and AF3 are remarkably accurate predictors of evolutionarily conserved protein–protein interactions, we used their multimer capabilities to search for significant contact points between the full-length, 3571 amino acid human SVEP1 chain and potential binding partners TIE1 and TIE2. As shown by the comparative PAE (Predicted Aligned Error, a prediction quality and model confidence metric) plots of the full-length SVEP1 matches with TIE1 and TIE2 (Figure 1A and B), we observe clustering between the headpiece or N-terminal D1-D4 domains of TIE1 and the CCP19-21 stretch of SVEP1, in line with the results of Sato-Nishiuchi et al., 2023, which center on the CCP20 domain of murine SVEP1 for TIE1 capture. D1 and D2 refer to the two N-terminal Ig domains, D3 refers to the three EGF domains, and D4 to the third Ig domain of TIE1 or TIE2. By contrast, the PAE plot for the D1-D4 segment of TIE2 fails to show an equivalent clustering with the CCP19-21 stretch of full-length SVEP1. The more focused interrogation and modeling of equivalent N-terminal TIE1 and TIE2 ecto-domains with the isolated CCP19-CCP21 domain fragment of SVEP1 (using AF2.3 multimer) affirms the interaction interface of SVEP1 to TIE1, positioning the groove between the side-by-side packed Ig-like D1-D2 domains of TIE1 in close contact with the CCP20 domain of SVEP1, revealing an interaction surface area of 851.5 Å^2^ marked by eight H-bonds and two salt bridges by PDBePISA analysis (Figure 1—figure supplement 1). The SVEP1-binding domains of TIE1 are buttressed by a Cys-rich D3 domain that links to an outstretched D4 Ig module that further connects to a linear array of D5-D7 FnIII domains N-terminal to the hydrophobic TM helix. Key regions and contacts illustrating salt bridges, hydrogen bond network surface, electrostatic potential representation, and hydrophobicity surface representations are shown in Figure 1—figure supplement 2. In this closer examination of molecular fragments of TIE2 and SVEP1, AF2.3 multimer scores the interface lower in both ipTM (interface predicted template modeling) and actifpTM (actual interface predicted template modeling) metrics (Figure 1—figure supplement 1) and qualitatively weaker binding to the CCP19-21 fragment of SVEP1. The resulting SVEP1-TIE2 model shows the D1 and D2 domains of TIE2 perched between the CCP20 and CCP21 domains of SVEP1 (Figure 1B). While PDBePISA analysis shows a similar interface surface area of 889.4 Å^2^, only two H-bonds and one salt bridge are observed, suggesting a weaker interface. To verify the modeling results, we performed SPR assays with a 70 kDa version of the SVEP1 protein that contains the CCP20 domain (Figure 1C). We observed interaction of SVEP1 with TIE1, but not with TIE2 in the absence of other proteins. Furthermore, utilizing negative staining and transmission electron microscopy, we were able to directly visualize the attachment of SVEP1 and TIE1 (Figure 1D). As shown in Figure 1—figure supplement 2, modeling of SVEP1 and TIE1 suggested P202 and L203 of TIE1 as a key contact region. Accordingly, a TIE1 (P202L-L203F) mutant was not able to bind to SVEP1 (Figure 1—figure supplement 3B). In addition and in agreement with a previous study, a SVEP1 (E2568A-G2569A) mutant in which two crucial amino acids in the CCP20 domain are exchanged could not bind to TIE1 (Figure 1—figure supplement 3A).

**Figure 1—figure supplement 1.**
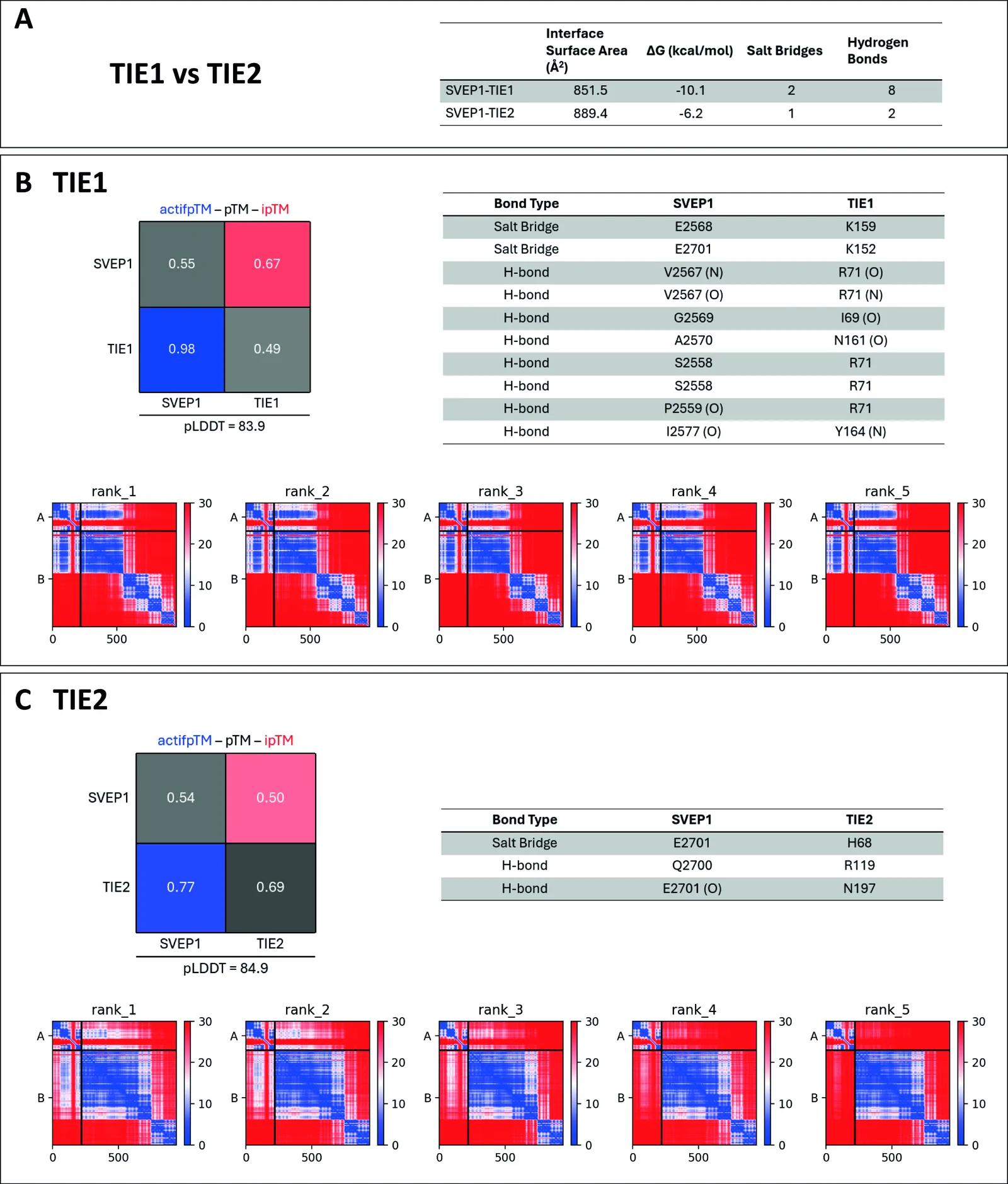
Alphafold confidence plots and scores, characterization of the physical interface of SVEP1/TIE1 or SVEP1/TIE2 complexes. Statistics and (**A**) PDBePISA analysis of a SVEP1-TIE1 ectodomain model vs. a SVEP1-TIE2 ectodomain model depicting interface surface area, Gibbs free energy, salt bridges, and hydrogen bond contacts. (**B, C**) Statistics table, bonds, and PAE plot of TIE1 (**B**) or TIE2 (**C**). PAE plot: A = SVEP1 CCP19-21, B=TIE1 or TIE2.

**Figure 1—figure supplement 2.**
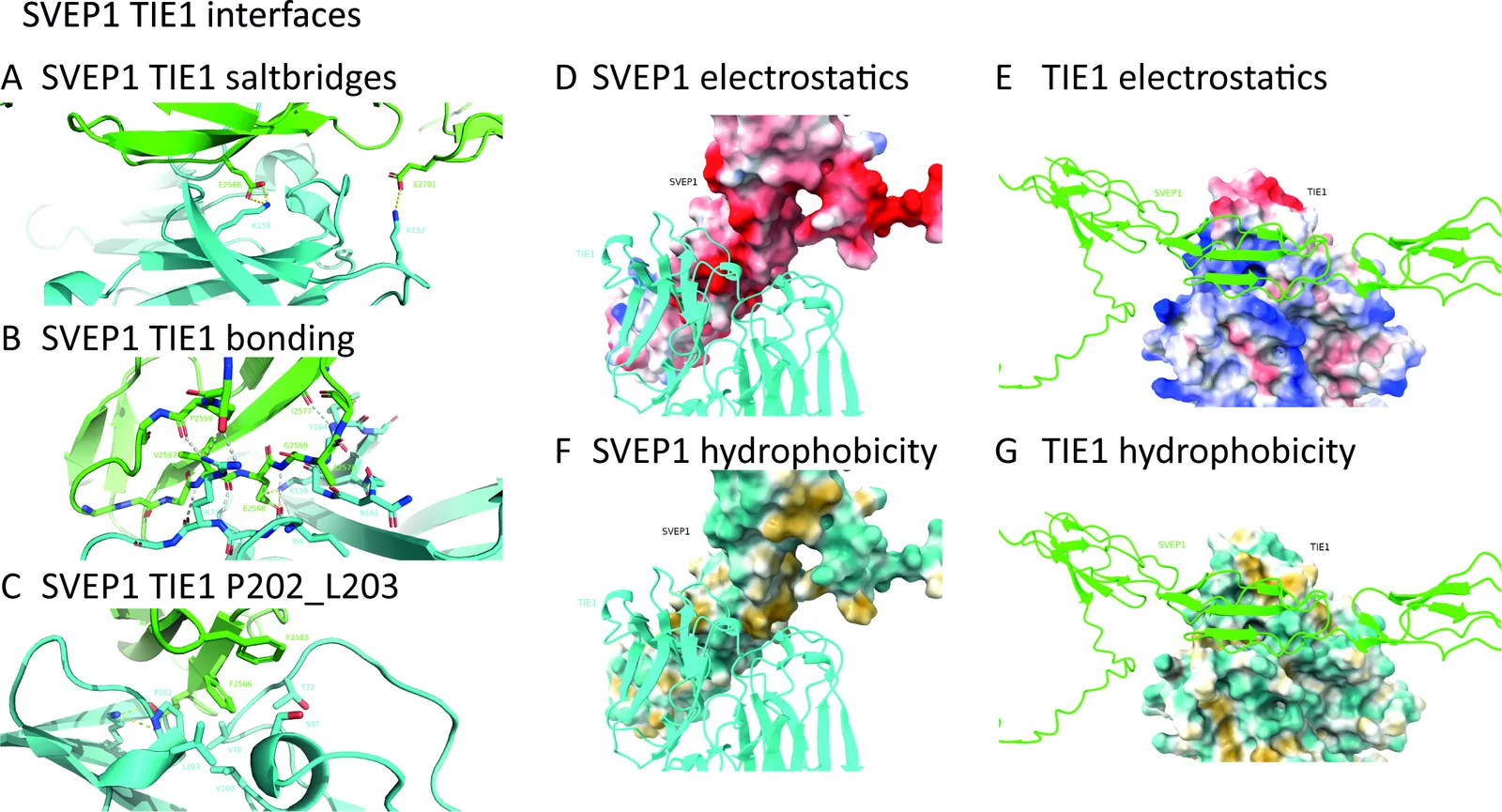
SVEP1 TIE1 interfaces. (**A**) Salt bridges E2568-K159 and E2701-K152 between SVEP1 CCP20 (green) and D1/D2 of TIE1 ectodomain (cyan). Electrostatic bonds are highlighted in yellow. (**B**) Hydrogen bond network of SVEP1 CCP20 (green) and D1/D2 of TIE1 ectodomain (cyan). H-bonds are highlighted in white. (**C**) F2566 of SVEP1 (green) fits in the hydrophobic pocket of TIE1 (cyan) partially formed by P202 and L203. (**D, E**) Surface electrostatic potential representation of SVEP1 (green) bound to TIE1 (cyan) detailing that the mainly acidic interaction surface of SVEP1 (**D**) is opposed by a basic interaction surface of TIE1 (**E**). (**F, G**) Hydrophobicity surface representations of SVEP1 (green) bound to TIE1 (cyan). The hydrophobic side chains of F2566 and F2583 fit into a hydrophobic cavity of TIE1.

**Figure 1—figure supplement 3.**
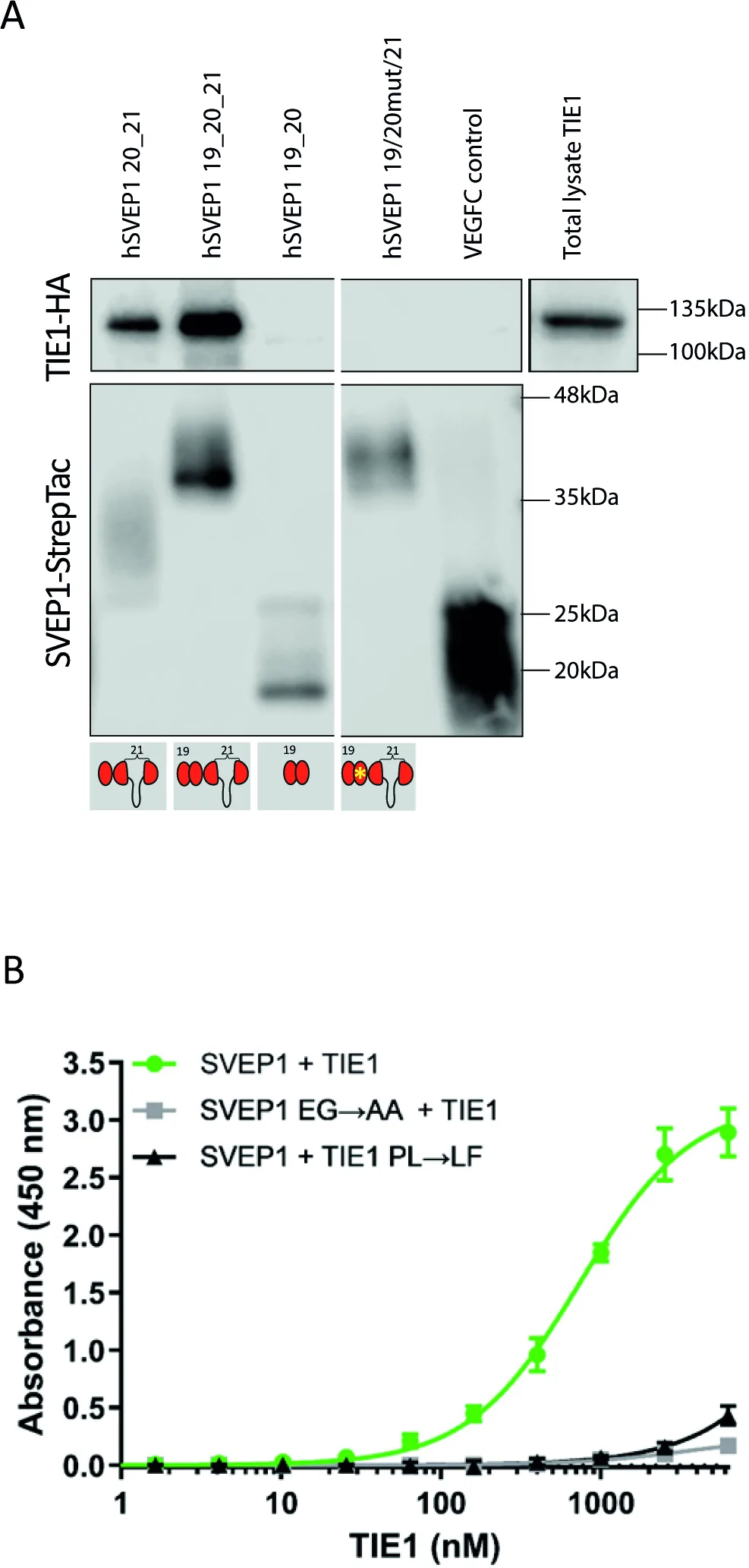
SVEP1 (E2568 - G2569) and TIE1 (P202 - L203) are required for SVEP1/TIE1 binding. (**A**) Different versions of human SVEP1-StrepTac (spanning from CCP15 to the C-Term) were immunoprecipitated and associated TIE1 was detected via Western blot analysis. TIE1 bound to CCP19-21 as well as CCP20-21, but not the CCP19-21 mutant containing the E2568A-G2569A mutant or CCP19-20. (**B**) Binding of SVEP1 (wt or E2568A-G2569A) to TIE1 (wt or P202L L203F) was analyzed by ELISA. Both mutations, either in TIE1 or in SVEP1, inhibit binding between TIE1 and SVEP1. Figure 1—figure supplement 3—source data 1.This source data contains the original files of the full raw uncropped, unedited blots presented in Figure 1—figure supplement 3. Figure 1—figure supplement 3—source data 2.This source data contains figures with the uncropped gels or blots presented in Figure 1—figure supplement 3.

### ANG1/2 strengthens the binding capacity of SVEP1 and TIE1

**Figure 2.**
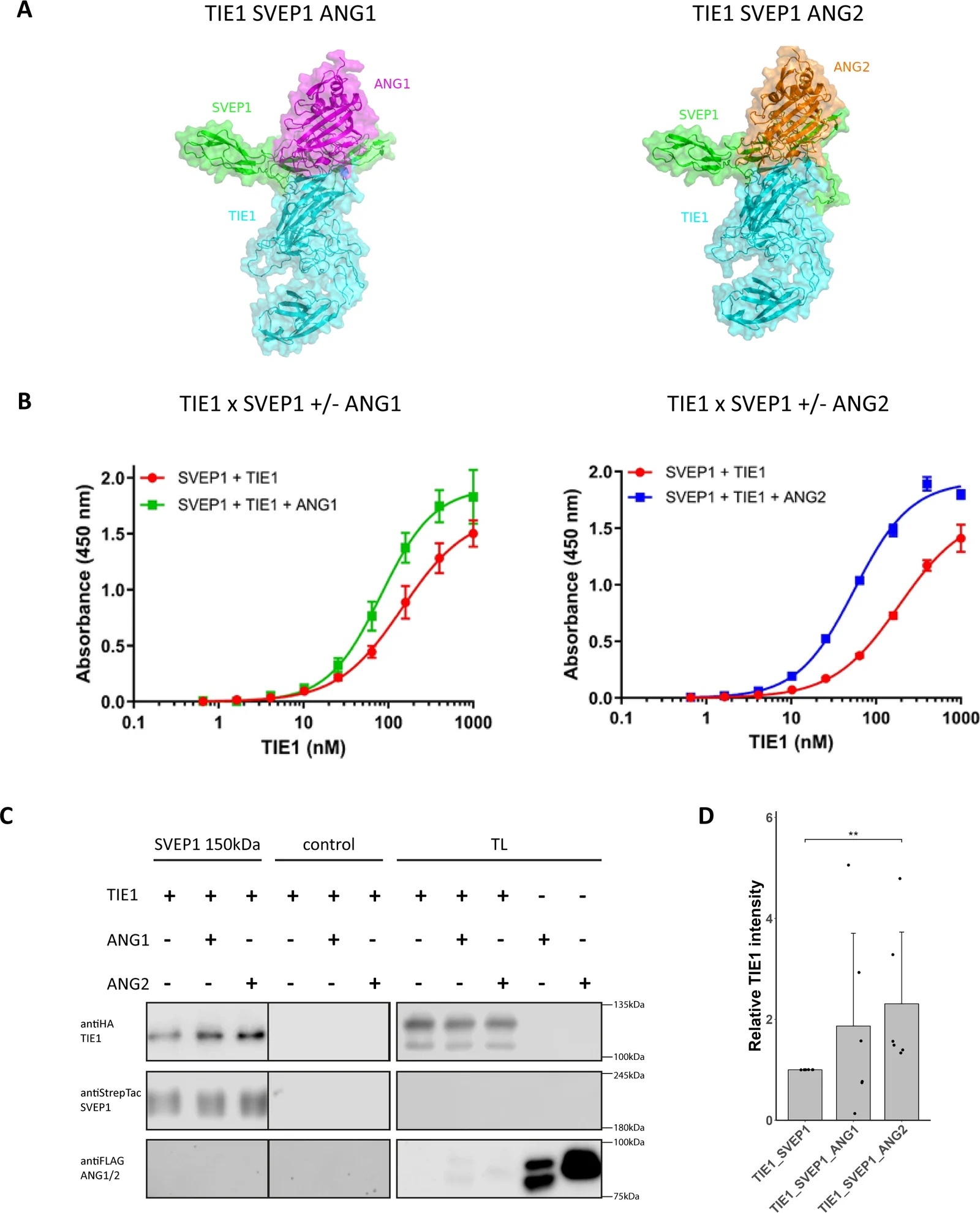
ANG1/2 strengthens the binding capacity of SVEP1 and TIE1. (**A**) AF2.3-modeling of the SVEP1 CCP19-21 domain and TIE1 together with ANG1/2. The affinity of TIE1 for SVEP1 is considerably heightened by the co-binding of ANG1 or ANG2 C-terminal domains to the CCP20 module. (**B**) The interaction between the complete C-terminal SVEP1 (spanning from the EGF1 domain to C-terminus) and the ectodomain of TIE1 was analyzed by ELISA binding assays. SVEP1 proteins were immobilized on 96 well plastic plates and overlayed with serial dilutions of TIE1 fused to an ALFA-tag, in the absence or presence of equal amounts of ANG2 as indicated. Bound TIE1 proteins were detected by HRP conjugated anti-ALFA-tag nanobody. ANG2 binding was detected using an HRP conjugated anti-Biotin antibody against the biotinylated protein. KD values are lower in the presence of ANG1 (K_D_ = 82,4) or ANG2 (K_D_ = 55,6), reflecting a higher affinity than SVEP1 and TIE1 in the absence of angiopoietins (K_D_ = 189,0). (**C**) Co-immunoprecipitation of human TIE1 co-transfected with either human ANG1 or ANG2 in 293T HEK cells. Human SVEP1-Strep II (a 150 kDa version reaching from CCP15 to the C-Term) was immunoprecipitated, and associated TIE1 and ANG1/2 were detected via Western blot analysis. (**D**) Quantification of the TIE1 levels with SVEP1 levels as a reference confirmed a significant increase in binding affinity of SVEP1 and TIE1 together with ANG2 (**p.adj=0.008; Mann–Whitney *U* test; Values are presented as means ± SD; individual data points for each experiment). TL, total lysate. Figure 2—source data 1.This source data contains the original files of the full raw uncropped, unedited blots presented in Figure 2. Figure 2—source data 2.This source data contains figures with the uncropped gels or blots presented in Figure 2. Figure 2—source data 3.This source data contains PDBs (.cif and .json files) of modelling of the SVEP1/TIE1/ANG1 complexes shown in Figure 2 and its figure supplements. Figure 2—source data 4.This source data contains PDBs (.cif and .json files) of modelling of the SVEP1/TIE1/ANG2 complexes shown in Figure 2 and its figure supplements.

Murine SVEP1 binds ANG1 and ANG2 in vitro (Morooka et al., 2017), and it has long been known that ANG1 and ANG2 bind to TIE2 (Davis et al., 1996; Maisonpierre et al., 1997). Both AF2 and AF3 multimer models effectively capture TIE1 in a complex with SVEP1 CCP20, so we next explored how the addition of ANG1/2 would affect this assembly (Figure 2A, Figure 2—figure supplements 1 and 2). The affinity of TIE1 for SVEP1 is significantly increased by the simultaneous binding of the C-terminal domains of ANG1 or ANG2 to the CCP20 module, as suggested by the increased PAE and ipTM scores, which improve on the binary coupling of just TIE1 to SVEP1 (Figure 2—figure supplement 1). By contrast (not shown), TIE2 was not predicted to stably co-bind with ANG1/2 to CCP19-CCP21 of SVEP1 because the weaker binding of TIE2 with CCP19-CCP21 of SVEP1 fails to nucleate the formation of a ternary complex. The conformation of the TIE1-ANG1/2 heteromers in the SVEP1-bound pose resembles the solved X-ray structures of TIE2 bound to either ANG1 or ANG2 (PDB codes 4K0V and 2GY7, respectively, in the absence of any experimental TIE1-ANG complexes) (Figure 2—figure supplement 1), and furthermore, the equivalent ANG1/2-binding epitopes in TIE1 are not blocked by SVEP1 binding. Whereas the two TIE2 X-ray complexes show a buried surface area of ~1200 Å^2^ (with two H-bonds and two salt bridges) against ANG1/2, the addition of the SVEP1-TIE1 contact produces a combined ~1800 Å^2^ interaction surface area as well as new contacts between SVEP1-ANG1 and SVEP1-ANG2 (three H-bonds and one H-bond, respectively) (Figure 2—figure supplement 1). For this reason, SVEP1’s CCP20 module acts as a binding partner for TIE1, but not TIE2, when interacting with ANG1/2.

**Figure 2—figure supplement 1.**
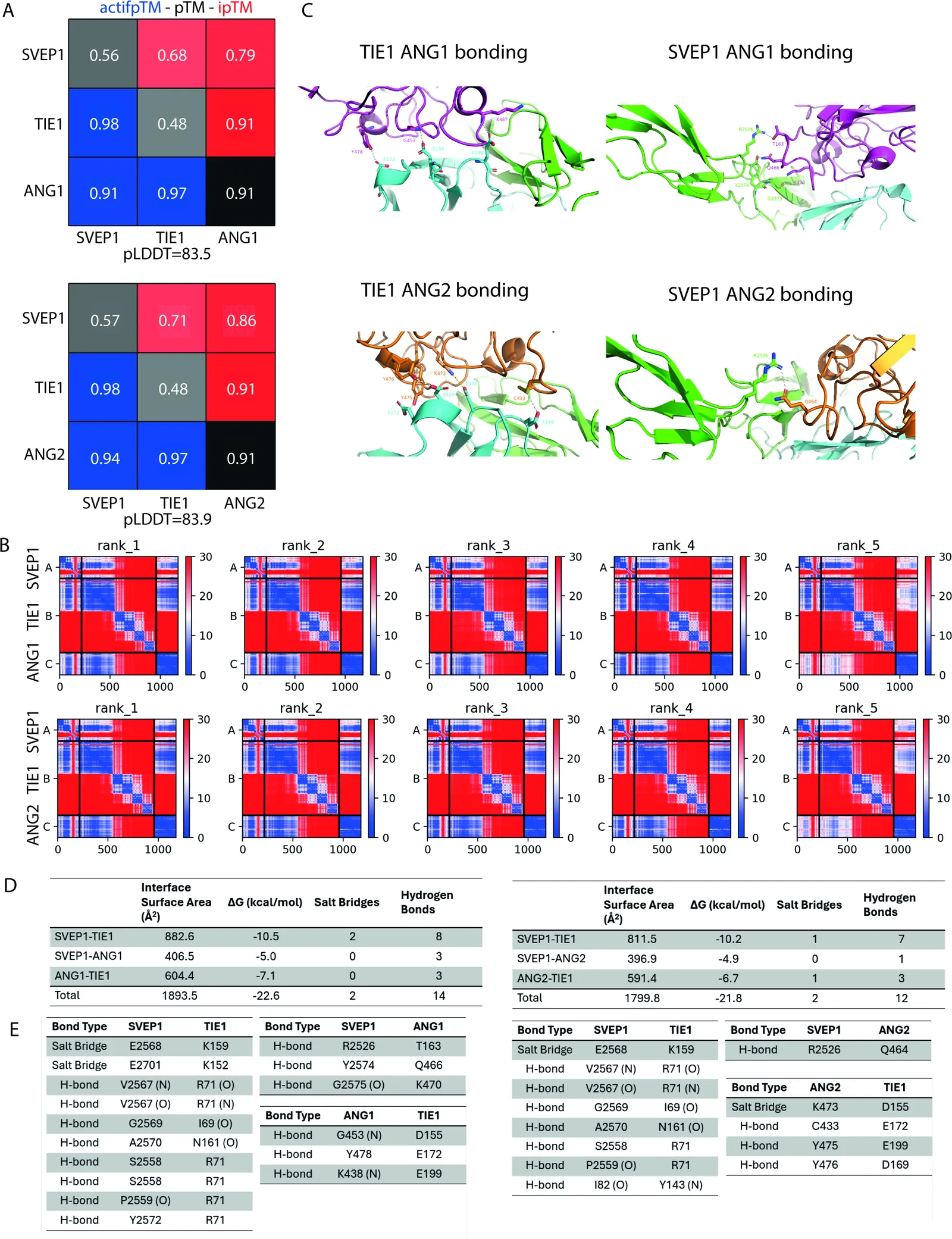
Alphafold confidence plots & scores and bonding of SVEP1/TIE1/ANG1 or SVEP1/TIE1/ANG2 complexes. (**A**) AlphaFold 2.3 multimer output scores and (**B**) PAE plots of SVEP1-TIE1-ANG1/2 complexes. A is SVEP1 CCP19-21, B is TIE1 ectodomain, and C is the ANG1/2 fibrinogen-like domain. (**C**) Bonding residues of each interface (**D**) and (**E**) PDBePISA analysis of interfaces of SVEP1-TIE1-ANG1/2 top ranked models. The conformation of the TIE1-ANG1/2 heteromers in the SVEP1-bound pose resembles the solved structures of TIE2 bound to either ANG1 or ANG2. However, the weaker binding of TIE2 to CCP19-21 of SVEP1 fails to nucleate the formation of the TIE2 ternary complex with SVEP1.

**Figure 2—figure supplement 2.**
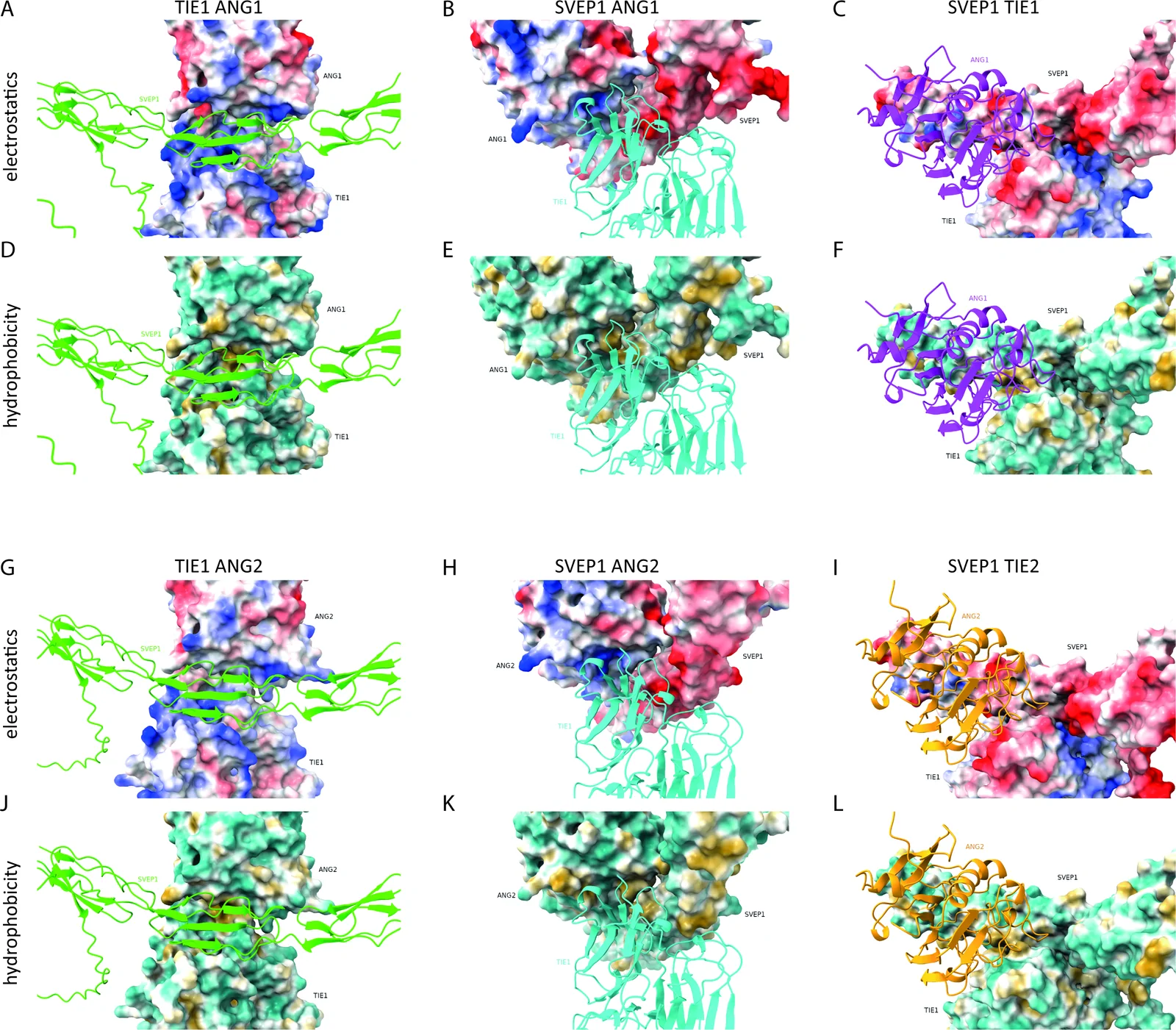
Interaction surfaces in SVEP1/TIE1/ANG1 or SVEP1/TIE1/ANG2 complexes. (**A–C**) Surface electrostatic potential of the interaction surfaces present in the SVEP1-TIE1-ANG1 complex demonstrating complementary charge distributions. (**D–F**) Surface hydrophobicity of the interaction surfaces present in the SVEP1-TIE1-ANG1 complex. (**G–I**) Surface electrostatic potential of the interaction surfaces present in the SVEP1-TIE1-ANG2 complex. (**J–L**) Surface hydrophobicity of the interaction surfaces present in the SVEP1-TIE1-ANG2 complex. All electrostatic potentials and hydrophobicity surfaces were generated and visualized with UCSF Chimera 1.19.

**Figure 2—figure supplement 3.**
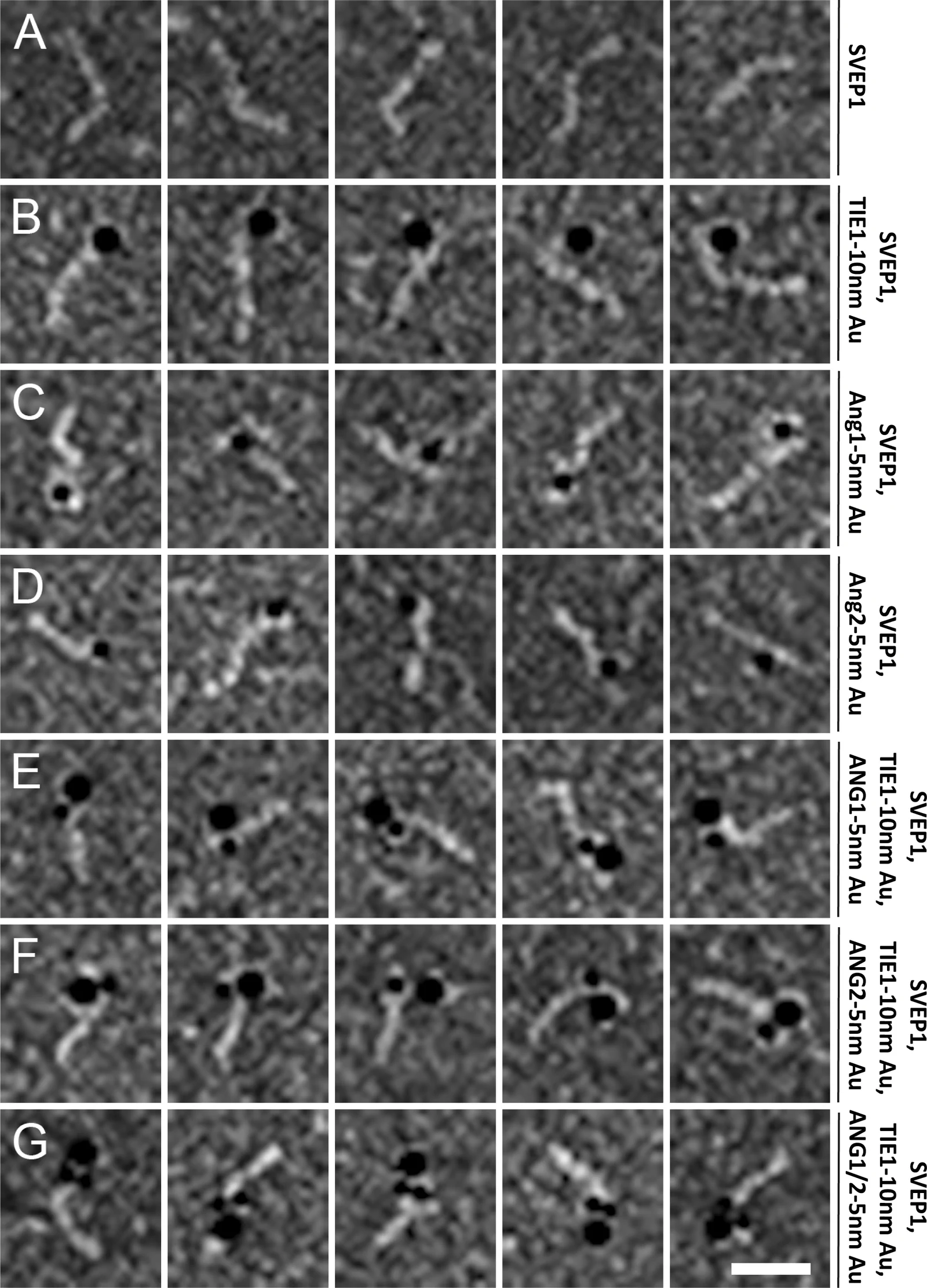
ANG1 and ANG2 bind in close proximity to the site where TIE1 and SVEP1 interact. Negative staining of human SVEP1 binding to TIE1 with and without the presence of ANG1 or ANG2. (**A**) hSVEP 150 kDa. (**B**) hSVEP-hTIE-10 nm Au. (**C**) hSVEP-ANG1-5nm Au, (**D**) hSVEP-ANG2-5nm Au. (**E**) hSVEP-hTIE-10 nm Au-ANG1-5 nm Au. (**F**) hSVEP-hTIE-10 nm Au-ANG2-5 nm Au. (**G**) hSVEP-hTIE-10 nm Au-ANG1/2-5 nm Au. Parts of panels (A) and (B) (control, SVEP1-TIE1-10 nm Au) have also been shown in main Figure 1. Scale bar: 25 nm.

ELISA assays verified the model prediction as the presence of either ANG1 or ANG2 led to a lower KD value (SVEP1, TIE1 (KD = 146,9) vs. SVEP1, TIE1, ANG1 (KD = 82,4); SVEP1, TIE1 (KD = 189) vs. SVEP1, TIE1, ANG2 (KD = 55,6)), reflecting a higher affinity (Figure 2B). Furthermore, co-transfection of either ANG1 or ANG2 together with TIE1 and subsequent immunoprecipitation of SVEP1 bound to beads verified the model prediction (Figure 2C). Whereas SVEP1 levels in all samples were similar, the level of TIE1 bound to SVEP1 significantly increased when ANG1/2 was present in the lysate. TIE1 co-transfected with ANG1 led to a slight, but not significant, increase in TIE1 levels. However, after transfection of TIE1, ANG1 and ANG2 levels are decreased in the total lysate. Thus, we were not able to detect ANG1 or ANG2 after immunoprecipitation. Furthermore, negative staining shows binding of TIE1 and ANG1/2 to SVEP1 (Figure 2—figure supplement 3).

### Stimulation by SVEP1 and ANG1/ANG2 leads to phosphorylation of TIE1, nuclear exclusion of FOXO1, and phosphorylation of AKT

**Figure 3.**
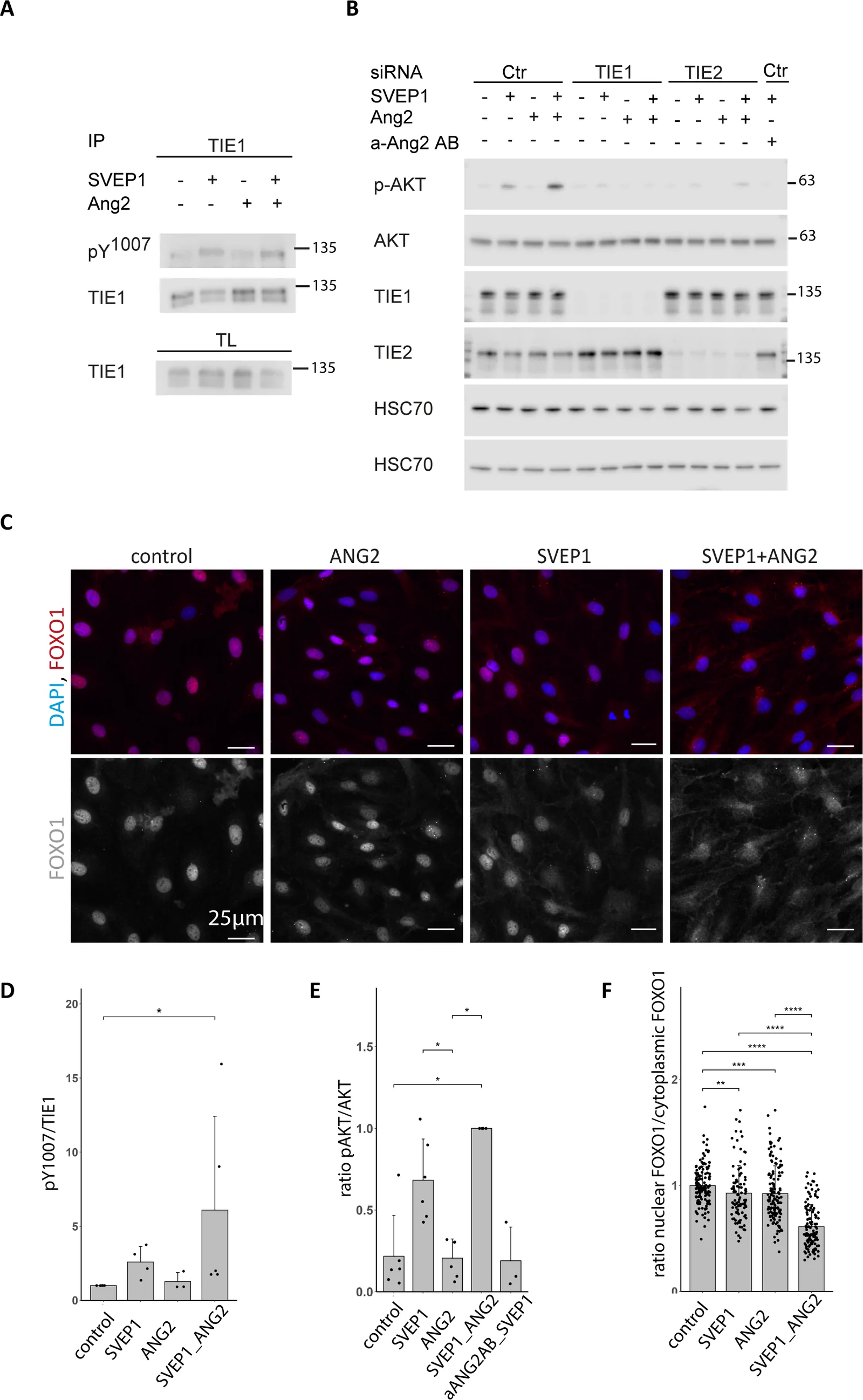
Simultaneous stimulation of LECs with SVEP1 and ANG2 leads to phosphorylation of TIE1 and AKT as well as nuclear exclusion of FOXO1 in vitro. (**A**) Western blot analysis of TIE1 phosphorylation at Y^1007^ residue in SVEP1 and/or ANG2 stimulated LECs after immunoprecipitation of TIE1. TIE1 is phosphorylated after simultaneous stimulation with SVEP1 and ANG2. The antibody anti-phospho-TIE2 (pY992, AF2720 R&D Systems) detects both TIE1 phosphorylation (at Y1007 residue) and TIE2 phosphorylation (at Y992 residue), but can be distinguished by size (Brouillard et al., 2024). (**B**) Western blot analysis of p-AKT and AKT in the cell lysate of SVEP1 and ANG2 stimulated LECs. Knockdown of either TIE1 or TIE2 leads to reduced induction of phosphorylation of AKT by SVEP1-ANG2 (**C**) FOXO1 (red) and DAPI (blue) staining of LECs stimulated with only SVEP1 or ANG2, or in combination. FOXO1 staining is restricted to the nucleus of unstimulated (control) LECs, whereas in cells stimulated with SVEP1 and ANG2 together FOXO1 is in the cytoplasm. (**D**) Quantification of the pTIE1/total TIE1 ratios shown in (A) (N=5 (control and SVEP1_ANG2), N=4 and 3 (SVEP1 and ANG2, respectively); *p adj = 0,045; Mann–Whitney *U* test; values are presented as means ± SD). (**E**) Quantification of the pAKT/total AKT ratios shown in (B). Values are presented as means ± SD, N=6 (control, SVEP1, ANG2, SVEP1_ANG2) or 3 (aANG2AB_SVEP1); Mann–Whitney *U* test; Control vs. ANG2_SVEP1 p.adj=0.028 *; SVEP1 vs. ANG2 p.adj=0.022 *; ANG2 vs. ANG2_SVEP1 p.adj=0.028 *; ANG2_SVEP1 vs. aANG2AB_SVEP1 p.adj=0.091. (**F**) The ratio of nuclear FOXO1/cytoplasmic FOXO1 decreases after stimulation with either ANG2 or SVEP1. Stimulation of LECs with simultaneous SVEP1 and ANG2 leads to a further reduction of the nuclear FOXO1/cytoplasmic FOXO1 ratio. Each datapoint represents the mean value per image of the ratio of nuclear to cytoplasmic FOXO1 per cell (Mann–Whitney *U* test; values are presented as means ± SD; N≥99 from 4 independent assays). Figure 3—source data 1.This source data contains the original files of the full raw uncropped, unedited blots presented in Figure 3. Figure 3—source data 2.This source data contains figures with the uncropped gels or blots presented in Figure 3.

In previous studies, phosphorylation of TIE1 could not be detected after stimulation of cells with SVEP1 (Sato-Nishiuchi et al., 2023) or ANG2 (Saharinen et al., 2005) only, whereas stimulation of EA.hy926 immortalized hybrid HUVECs with ANG1, ANG4, and COMP-ANG1 leads to phosphorylation of TIE1 (Saharinen et al., 2005). Our aim was to test whether cooperative binding of SVEP1 and angiopoietins to TIE1 can lead to TIE1 phosphorylation, where SVEP1 constitutes the connecting link between TIE1 and ANG2. Indeed, after stimulation of hdLECs with both SVEP1 and ANG2, robust phosphorylation of TIE1 at residue pY^1007^ was detected (Figure 3A and D). Also, stimulation with SVEP1 alone has a slight effect on the phosphorylation of TIE1, possibly because hdLECs express ANG2 (Korhonen et al., 2022). To further test whether SVEP1-ANG2 stimulation leads to downstream signaling, we analyzed pAKT levels. Stimulation with either ANG2 or SVEP1 led to phosphorylation of AKT, which was amplified by simultaneous stimulation with ANG2 and SVEP1. The effect of SVEP1 stimulation on AKT signaling could be blocked by an ANG2 blocking antibody, suggesting that SVEP1 does not induce signaling on its own, but enables signaling of ANG2 (Figure 3B and E). Knockdown of either TIE1 or TIE2 could reduce activation of pAKT, leading to the assumption that both TIE1 and TIE2 are necessary for the phosphorylation of AKT after SVEP1-ANG2 stimulation and thus downstream signaling. Furthermore, we analyzed FOXO1 nuclear exclusion, a downstream event of the PI3K/AKT pathway, by stimulating hdLECs with either ANG2 or SVEP1, or both proteins in combination, and testing for nuclear versus cytoplasmic FOXO1 ratios. It has already been shown that SVEP1 activates the PI3K-Akt signaling pathway in vitro, with nuclear exclusion of FOXO1 (Sato-Nishiuchi et al., 2023). We observed enhanced nuclear exclusion of FOXO1 in hdLECs stimulated with combined SVEP1 and ANG2 compared to ANG2 or SVEP1 alone (Figure 3C), indicating that ANG2 potentiates the activation of PI3K-Akt and thus inactivates FOXO1. ANG2 by itself had a comparable effect on the FOXO1 inactivation as SVEP1, with both proteins showing a significant effect (Figure 3F).

### SVEP1, TIE1, and ANG1/2 are predicted to form higher-order complexes

**Figure 4.**
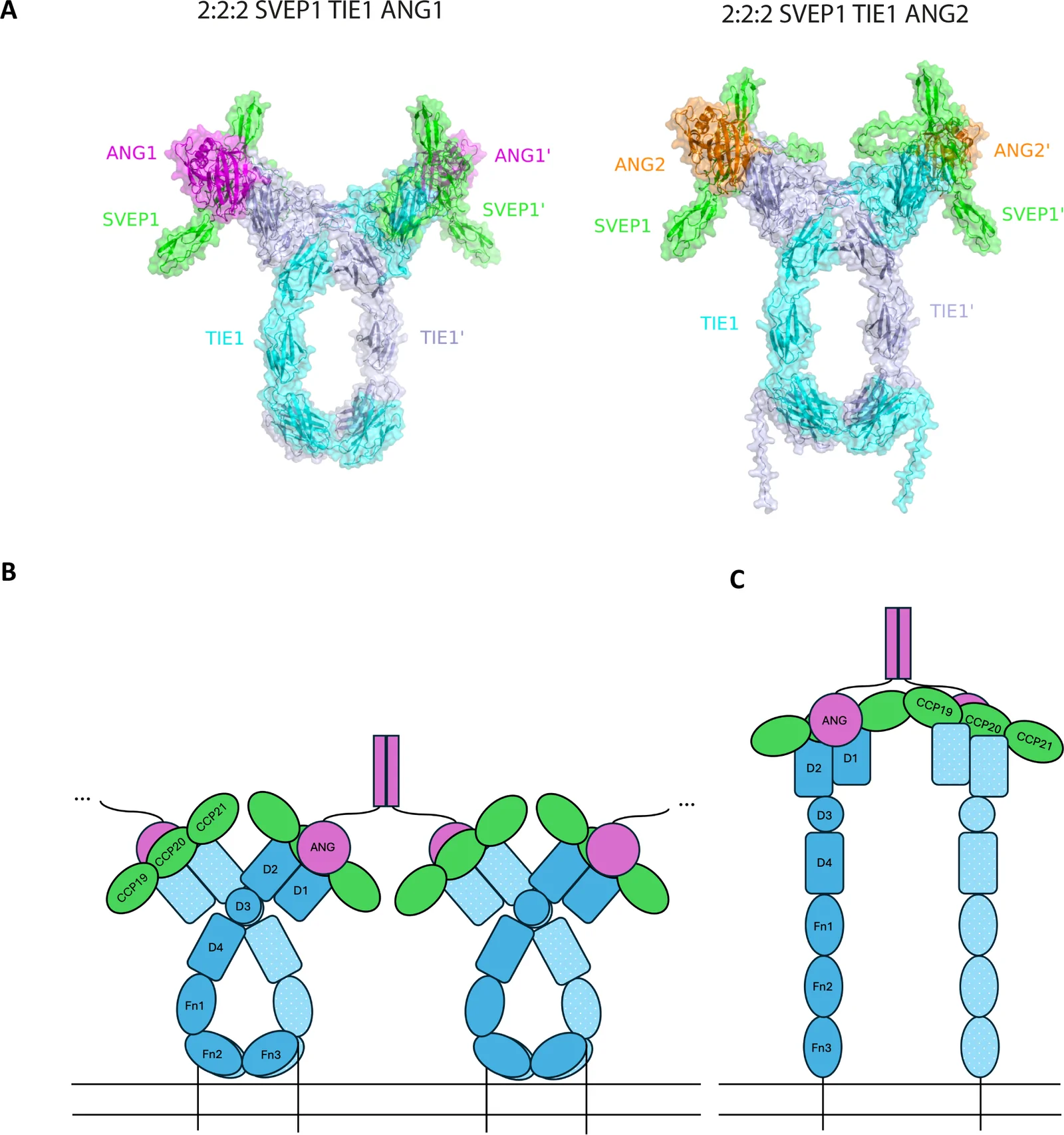
SVEP1, TIE1, and ANG1/2 can form 2:2:2 complexes mediated by SVEP1. (**A**) Larger hexameric complexes of SVEP1-TIE1 D1-D4-ANG1/2 in a 2:2:2 stoichiometry could be confidently modeled with AF3. They show a symmetric assembly linked by predicted TIE1 dimerization sites at FN3-FN2’ as well as the D3 and D4 domains. Similar to the 1:1:1 complex of SVEP1-TIE1-ANG1/2, ANG1/2 makes contact with the D1 and D2 domains of TIE1 by inclusion of the SVEP1 chain (CCP19-21). (**B**) Schematic model of SVEP1 enabling the multimerization of TIE1 receptors on the cell surface by cross-lacing of TIE1 dimers and N-terminal coiled-coil dimers (or greater assemblies) of ANG1/2. (**C**) Schematic model of TIE1 clustering via only ANG1/2-mediated multimerization. Figure 4—source data 1.This source data contains PDBs (.cif and .json files) of modelling of the 2:2:2 SVEP1/TIE1/ANG1 complexes shown in Figure 4 and its figure supplement. Figure 4—source data 2.This source data contains PDBs (.cif and .json files) of modelling of the 2:2:2 SVEP1/TIE1/ANG2 complexes shown in Figure 4 and its figure supplement.

The results show that SVEP1 together with ANG2 lead to phosphorylation of TIE1, which then activates the PI3K-AKT signaling pathway, leading to nuclear exclusion of FOXO1. Previous studies have demonstrated the importance of TIE2 receptor clustering and its impact on downstream signaling (Kim et al., 2009). To explore the ability of the SVEP1, TIE1, and ANG1/2 complexes to form higher order assemblies, we utilized AF3 to model a 2:2:2 complex. AF3 predicted that the previously modeled ternary complexes (Figure 2A) are incorporated into a larger assembly of dimerized TIE1 receptors (Figure 4A). In the presence of ANG1/2 and SVEP1, AF3 predicts that TIE1 dimerization is mediated by Fn3-Fn2’ contact as well as crossing over at the D3 and D4 domains (Figure 4B) with acceptable confidence metrics (Figure 4—figure supplement 1). In addition to TIE1 dimerization, both ANG1 and ANG2 dimerize via an N-terminal coiled-coil domain and can multimerize into larger assemblies by interchain disulfide bonds (Kim et al., 2005). ANG1/2 multimerization is crucial for proper TIE2 activation (Kim et al., 2009) and ANG1/2 together with SVEP1 may have a similar effect with TIE1, clustering TIE1 receptors, even if TIE1 does not dimerize on its own (Figure 4C). Both proposed models would position TIE1 receptors in close spatial proximity, thereby increasing the cytosolic signaling strength of the TIE1 kinase domains upon binding of SVEP1 and ANG1/2. While the models put forth are of good quality, further experimentation is needed to confirm the structural model of SVEP1, TIE1, and ANG1/2 multimerization proposed.

**Figure 4—figure supplement 1.**
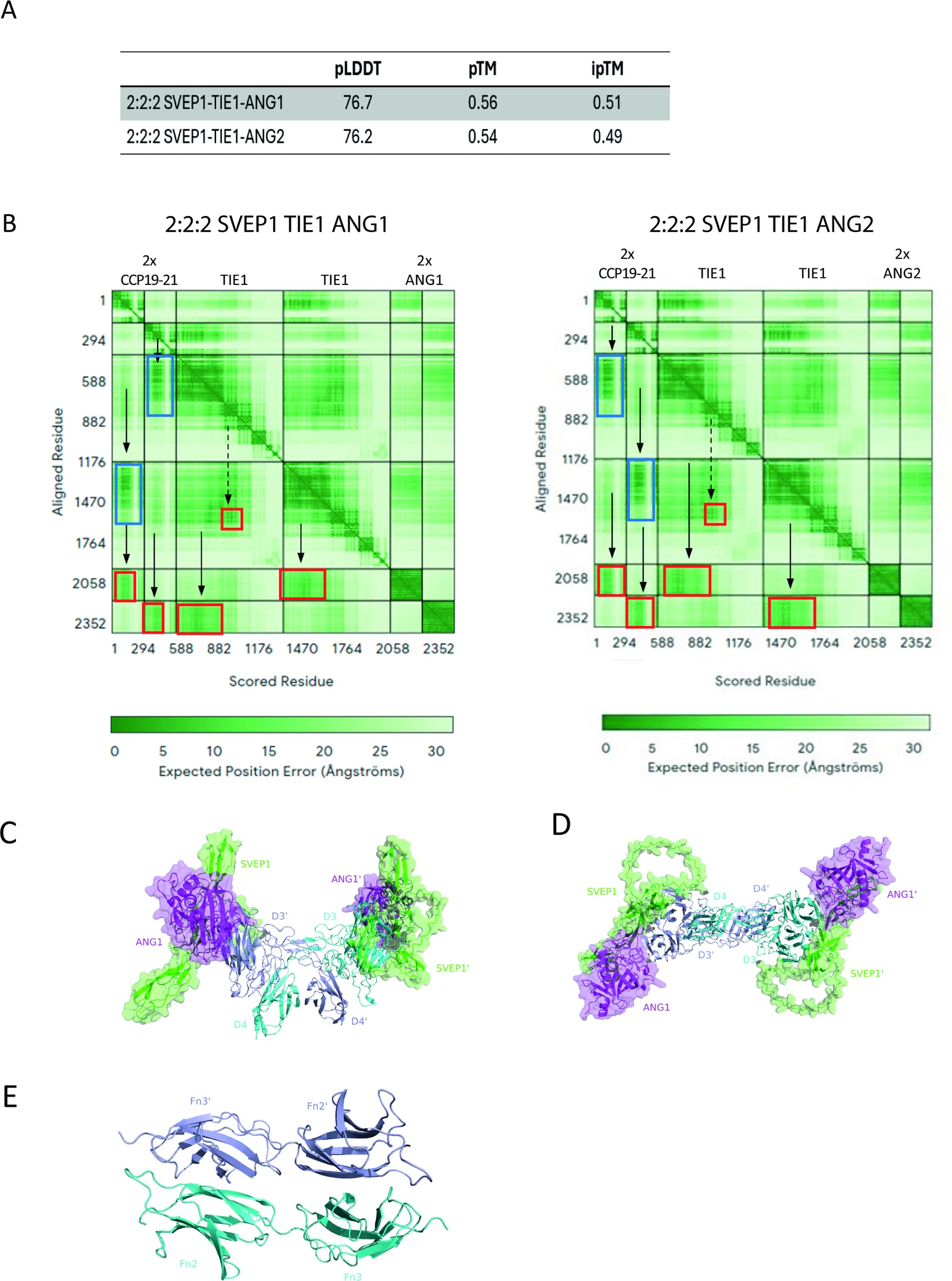
Confidence plots and scores and further views of the model hexamer. (**A**) AF3 output metrics. (**B**) PAE plots demonstrating confident predictions of the complexes shown in Figure 4. (**C, D**) The side (**C**) and top (**D**) views of the model hexamer show the side-by-side, antiparallel packing of TIE1 D3 domains that produces the domain-swapping of D4 Ig domains. (**E**) Top view of the Fn2–Fn3 domains.

## Discussion

TIE1 has long been considered an orphan receptor. Recently, we and others have shown that *svep1* and *tie1* genetically interact (Hußmann et al., 2023) and that SVEP1 binds to murine and human TIE1 (Hußmann et al., 2023; Sato-Nishiuchi et al., 2023). Here, we extend the analysis of SVEP1 as a binding ligand for TIE1 with the assistance of AF3 modeling and experimental support that demonstrates potentiated recruitment of TIE1 by SVEP1 in the presence of angiopoietins.

It is known that TIE2 is activated through binding of angiopoietins, including ANG1 and ANG2 (Davis et al., 1996; Maisonpierre et al., 1997), and that in humans and mice, angiopoietins do not directly bind to TIE1. In zebrafish, however, Ang1 binds to Tie1, Tie2 mutants do not have an obvious cardiovascular phenotype, and the *tie2* locus has been lost in many teleost species (Jiang et al., 2020). Therefore, zebrafish differ from humans and mice in terms of the TIE pathway, as zebrafish appear to exclusively use Tie1 and not Tie2 (Gjini et al., 2011; Jiang et al., 2020; Morooka et al., 2024). Both SPR testing and AF3 modeling revealed that SVEP1 (CCP15-CCP24) does not interact with TIE2. This is in line with trans-well migration assays, which revealed that SVEP1 induces the migration of lymphatic endothelial cells through binding to TIE1, but not TIE2 (Sato-Nishiuchi et al., 2023). Whether TIE2 is able to bind SVEP1 in a condition where other proteins like angiopoietins are present is still a question of interest and requires further investigation.

We here demonstrate via AF3 model and co-immunoprecipitation that the affinity of TIE1 for SVEP1 is highly enhanced by the simultaneous binding of the C-terminal domains of ANG2 to the CCP20 module, thus generating a complex of SVEP1, angiopoietins, and TIE1 molecules in which the angiopoietins are also able to bind TIE1. Utilizing AF3 modeling, we were able to confidently predict an interaction hub with a 2:2:2 complex of SVEP1, TIE1, and ANG1/2, where ANG1/2 binds different SVEP1 molecules by cross-lacing of N-terminal coiled-coil dimers, which then brings several TIE1 receptors into spatial proximity on the cell surface. This could lead to a cumulative increase in the signal strength of the cytosolic kinase TIE1. While TIE2 has been shown to dimerize via Fn3-Fn3’ contacts (Moore et al., 2017), evidence of TIE1 dimerization is limited. Leppänen et al., 2017 captured a strand swapped dimer of TIE1 Fn3 by X-ray crystallography (PDB: 5N06). However, solution SAXS data indicated monomeric TIE1 Fn3, leading to the conclusion that the observed crystallography dimer is likely to be a crystal artifact. However, our model as well as experimental data predicts that in the presence of SVEP1 and ANG1, TIE1 can dimerize and thus is phosphorylated upon ligand binding. So far, phosphorylation of TIE1 has not been detected when cells were stimulated with either SVEP1 or ANG2 only (Saharinen et al., 2005; Sato-Nishiuchi et al., 2023; Savant et al., 2015). Sato-Nishiuchi et al., 2023 could not detect an increase in the baseline phosphorylation of murine TIE1 in either LECs or 293-F cells that were treated with Svep1. Only TIE2 co-expression increased TIE1 phosphorylation irrespective of the presence or absence of Svep1. In blood and lymphatic endothelial cells, only COMP-Ang1 and ANG1 have been shown to stimulate the phosphorylation of TIE1 (Saharinen et al., 2005; Savant et al., 2015; Yuan et al., 2007). Co-transfection of TIE1 and TIE2 enhances TIE1 activation and phosphorylation due to heteromeric TIE1-TIE2 complexes. On the contrary, TIE2 phosphorylation is not enhanced by the presence of TIE1 (Saharinen et al., 2005), and TIE1 is not phosphorylated in the absence of TIE2 upon ANG1 stimulation (Savant et al., 2015). According to current knowledge, the phosphorylation of TIE1 is therefore dependent on TIE2. In zebrafish, however, it was shown that Tie1 can be phosphorylated (Morooka et al., 2024) and that Tie1, not Tie2, is the relevant receptor for lymphangiogenesis during early development. Furthermore, it was reported that autophosphorylation of TIE1 (Y1007) is not dependent on TIE2 but on TIE1, because substitutions in the kinase domain of TIE1 result in a loss of its baseline phosphorylation (Brouillard et al., 2024). Additionally, after COMP-Ang1 stimulation of TIE1-transfected 293T cells that do not express TIE2, weak phosphorylation is observed, whereby the activation is mediated by ANG1 and not by the COMP domain, a minimal coiled-coil segment that oligomerizes the ANG1 C-term module (Saharinen et al., 2005), which suggests that the phosphorylation is not dependent on TIE1/TIE2 heterodimerization. Nevertheless, since TIE1 was considered an orphan receptor during that time, the result could not be explained in 2005, and it was speculated whether COMP-Ang1-induced Tie1 activation could involve complex formation with additional molecules.

Our results show phosphorylation of TIE1 after combined SVEP1-ANG2 stimulation of hdLECs. SVEP1 is not expressed by HUVECs and LECs, but in non-endothelial cells (such as fibroblasts) in close proximity to LECs (Karpanen et al., 2017; Hußmann et al., 2023; Wang et al., 2020). According to data from the human protein atlas (https://www.proteinatlas.org), SVEP1 is also not expressed in HEK293 cells. Since SVEP1, which is necessary for the phosphorylation of TIE1, is provided by cells in the immediate vicinity of ECs and not by ECs themselves, the phosphorylation of TIE1 after ANG2 stimulation was never detected in cell culture experiments without SVEP1 being present. Whether TIE2 is necessary for the phosphorylation of TIE1 after SVEP1-ANG2 treatment needs further investigation. Our model shows that after binding of SVEP1 to TIE1, ANG1/2 are then able to dock onto this complex and serve as a ‘molecular glue’ for both SVEP1 and TIE1. Thus, TIE1-bound SVEP1 would serve as a cell-surface-tethered interaction hub for angiopoietins that are oligomerized—by either the native coiled-coil extensions or the minimal COMP domain—and create cross-linked fields of signaling TIE1 receptors (Figure 4).

The PI3K/Akt signaling pathway is involved in Svep1-induced LEC migration, as LECs show an increased Akt phosphorylation after Svep1 treatment (Sato-Nishiuchi et al., 2023). We were able to show that combined SVEP1-ANG2 stimulation leads to an even higher phosphorylation of AKT in human LECs than the sum of SVEP1 or ANG2 stimulations alone. Blocking of ANG2 using a function-blocking antibody prevents AKT phosphorylation after stimulation with SVEP1, implying that SVEP1 cannot induce downstream signaling on its own, but only in combination with angiopoietins.

Furthermore, both TIE1 and TIE2 are required for the full signaling potential of SVEP1-ANG2, as knockdown of either TIE1 or TIE2 reduced the phosphorylation of AKT upon SVEP1-ANG2 stimulation. A signaling event downstream of PI3K/Akt is the inactivation and nuclear exclusion of FOXO1 (Daly et al., 2004), which is reduced in Tie1-silenced vascular endothelial cells (Korhonen et al., 2016). Furthermore, SVEP1 induces FOXO1 nuclear exclusion. This was shown in LECs as well as in situ where FOXO1 immunostaining of lymphatic vessels in wild-type mice was detected in the nucleus, whereas in *Svep1*-deficient mice, FOXO1 staining was detectable in both the nucleus and cytoplasm (Sato-Nishiuchi et al., 2023). During secondary sprouting of endothelial cells in the zebrafish trunk, Tie1 induces nuclear exclusion of *foxo1a,* since *tie1* mutants displayed an enhanced localization of *foxo1a* in the nucleus (Morooka et al., 2024). Thus, the nuclear versus cytoplasmic ratio of FOXO1 is used as a readout for signaling in a number of systems. Here we show that stimulation of LECs with a combination of SVEP1 and ANG2 leads to a stronger nuclear exclusion of FOXO1 than with either factor alone, thus indicating that stronger binding of SVEP1 to TIE1 also potentiates downstream signaling of TIE1.

The CCP20 module of SVEP1 acts as a critical binding partner for TIE1, but not TIE2, when interacting with ANG1/2. However, it remains unclear whether TIE2 could bind to other sites of SVEP1 in the presence of other proteins, since in the SPR assay and the immunoprecipitation assays, only part of the C-terminus of the SVEP1 protein was used. Also, TIE1 but not TIE2 is required for SVEP1-induced migration (Sato-Nishiuchi et al., 2023), but both are necessary for SVEP1-stimulated phosphorylation of AKT. Furthermore, in capillaries, only depletion of *Tie1*, but not of *Tie2,* had an effect, whereas in collecting vessels, depletion of *Tie2* resulted in a phenotype (Korhonen et al., 2022). This result can most likely be explained by high expression levels of TIE2 in collecting vessels, but low expression levels of TIE2 in capillaries. Additionally, it is proposed that SVEP1 acts as a modifier of TIE2 expression during Schlemm’s canal development as patients that are trans-heterozygous for TIE2 and SVEP1 develop glaucoma and the p.R997C-SVEP1 variant, which is mutated in some glaucoma patients and located shortly after the Furin cleavage site, fails to enhance TIE2 expression in HEK293T cells compared to wild-type SVEP1 (Young et al., 2020). Depending on the downstream signaling event as well as the lymphatic cell type (capillaries versus collecting vessels), TIE2 might be needed for phosphorylation of TIE1 either independently of SVEP1 or for binding to other domains of SVEP1 than CCP19-21.

The activation mechanisms of the TIE receptors have long been a point of contention. Especially TIE1 activation and how possible signaling occurs has remained enigmatic, and only recently SVEP1 has been demonstrated to serve as a ligand for the TIE1 transmembrane receptor, which for many years had been considered an orphan receptor with phosphorylation believed to be dependent on TIE2. We here show that SVEP1-ANG2 is able to cause phosphorylation of TIE1 and show that the presence of ANG2 results in stronger recruitment of TIE1 to SVEP1, supporting a model that predicts a higher-order complex of SVEP1, TIE1, and ANG1/2. In such complexes, ANG1/2 binds different SVEP1 molecules by cross-lacing of N-terminal coiled-coil dimers, which then brings several TIE1 receptors into spatial proximity on the cell surface. This could lead to the formation of a signaling hub, where the signal strength of the cytosolic kinase TIE1, or another signaling moiety, is increased.

## Materials and methods

### Antibodies and reagents

#### Recombinant protein expression

Recombinant proteins (SVEP1, GenBank: NM_153366.5), (ANG1, GenBank: NM_001199859), (ANG2, GenBank: NM_001118887), TIE1 (GenBank: NM_005424), and TIE2 (GenBank: NM_000459) were amplified from human cDNA by PCR. Amino acids contained in the different recombinant proteins are listed in the ‘key resources table’. Protein production was carried out essentially as described (Hußmann et al., 2023; Meier et al., 2023). Briefly, SVEP1, TIE1, and ANG2 containing an N-terminal twin Strep-tag were expressed in HEK 293T cells using the inducible sleeping beauty transposon system and secreted into the medium. Tagged proteins were bound to Strep-Tactin XT 4Flow matrix (IBA Lifescience) equilibrated in 50 mM Tris pH 8.0, 150 mM NaCl. After washing with 50 mM Tris pH 8.0, 1 M NaCl proteins were recovered using Biotin-containing BXT elution buffer (IBA Lifescience) and subsequently dialyzed into PBS. PCR products were cloned into a modified sleeping beauty transposon expression vector containing a BM40 signal peptide sequence and a Twin-Strep-tag. For recombinant protein production, stable HEK293 EBNA cell lines were generated employing the sleeping beauty transposon system (Kowarz et al., 2015). Briefly, expression constructs were co-transfected with a transposase plasmid (1:10) into the HEK293 EBNA cells using FuGENE HD transfection reagent (Promega). After selection with puromycin (2 µg/ml), cells were induced with doxycycline. Supernatants were filtered and the recombinant proteins purified via Strep-TactinXT (IBA Lifescience) resin. Proteins were then eluted by biotin-containing TBS-buffer (IBA Lifescience) and subsequently dialyzed into PBS. The human sequences of *ANGPT1* (corresponding to NM_001199859, aa: 20–497) and *ANGPT2* (corresponding to NM_001118887, aa: 20–495) were cloned into the PCEP episomal expression system (transient), including an N-terminal Flag-tag sequence.

#### Surface plasmon resonance

Protein–protein interactions were analyzed by SPR experiments using a Biacore T200 system (Cytiva, Uppsala, Sweden). Human active SVEP1-Strep II recombinant protein, reaching from CCP15 to the CCP24, aa 2261–2890 and TIE1 (ectodomain TIE1, aa: 21–760) or TIE2 (ectodomain TIE2, aa: 23–745) were used. For immobilization of hSVEP1, a CM5 sensor chip was activated with N-hydroxysuccinimide and 1-ethyl-3-(3-dimethylaminopropyl) carbodiimide hydrochloride according to the manufacturer’s instructions, and 2050 response units (RU) of SVEP1 were coupled in 10 mM sodium acetate buffer, pH 4.0, at a flow rate of 10 µl/min. For the interaction measurements, the analytes, TIE1 and TIE2, were passed over the sensor chip at a flow rate of 30 µl/min in running buffer (10 mM HEPES, 150 mM NaCl, and 0.005% Tween20). The concentrations for the TIE proteins were 200 nM, 100 nM, 50 nM, 25 nM, 12.5 nM, 6.25 nM, and 3.125 nM. The binding kinetics were calculated by fitting a 1:1 interaction model using the BIAevalution software (Cytiva).

#### Negative staining

The interaction of the SVEP1 C-terminus with angiopoietin-1 was visualized by negative staining and transmission electron microscopy. Briefly, the proteins were incubated for 1 hour at 37°C in Tris-buffered saline (TBS), pH 7.4. Prior to the visualization in the electron microscope, the proteins were negatively stained with 1% uranyl acetate on the grids. In some experiments, the proteins were directly labeled with 5 nm (Sigma-Aldrich 752568) and 10 nm (Sigma-Aldrich 752584) colloidal gold, respectively, as previously described (Baschong and Wrigley, 1990). Specimens were examined in a Philips/FEI CM 100 TWIN transmission electron microscope operated at 60 kV accelerating voltage. Images were recorded with a side-mounted Olympus Veleta camera with a resolution of 2048×2048 pixels (2k × 2K) and the ITEM acquisitions software.

#### ELISA binding assays

ELISA assays were performed as described (Eble, 2018; Meier et al., 2023). Briefly, SVEP1 or SVEP1 mutants (2 µg/ml in TBS) were coated on 96-well plates (Nunc MaxiSorp, 50 µl/well) for 1 hour at room temperature and subsequently washed with TBS. Plates were blocked using Pierce Protein-Free Blocking Buffer (Thermo Fisher Scientific) for 1 hour at room temperature followed by 1% BSA in TBS overnight at 4°C. Bound SVEP1 was overlaid with serial dilutions of TIE1 in the presence or absence of ANG2, or TIE mutant containing an ALFA tag, or biotinylated ANG2 protein, and incubated for 1 hour at room temperature. After washing with PBS, protein complexes were fixed with 2.5% glutaraldehyde in PBS for 10 minutes and the reaction stopped by washing with TBS. Bound TIE or ANG2 proteins were detected with HRP conjugated anti-ALFA-tag nanobody or anti-Biotin antibody and stained using 1-Step Ultra TMB-ELISA (Thermo Fisher Scientific). After addition of 10% sulfuric acid, absorbance reading at 450 nm was done on a Tecan plate reader equipped with Magellan Pro software. Nonlinear regression analysis of data was performed using GraphPad Prism 11 software.

#### Cell culture and treatment/stimulation

HEK293T cells were cultured in DMEM (Thermo Fisher Scientific #61965026) and seeded 1 day before transfection utilizing Lipofectamine 2000 (Thermo Fisher Scientific, #11668027). hdLECs (Human Dermal Lymphatic Endothelial Cells, PromoCell) were cultured in Endothelial Cell Growth Medium MV2 (PromoCell, #C-22022) on Fibronectin-coated (Sigma, #F1141-1MG, 1 ug/ml) culture flasks. LEC culture medium was supplemented with 25 ng/ml VEGF-C (R&D, #9199-VC). Cells were seeded on ibidi slides (ibidi, #81201) and grown to confluency before the treatment.

For knock-down experiments, hdLECs were transfected with *TIE1*, *TIE2,* or control siRNA (see ‘key resources table’) using Lipofectamine RNAiMAX Transfection Reagent 1 day after splitting and 2 days prior to analysis of AKT phosphorylation.

For the pull-down assay, different versions of human Strep II tagged SVEP1 protein and a Strep II tagged control protein were incubated with 25 µl Strep-TactinXT 4Flow high-capacity resin (iba-lifesciences, #2-5030-002) in 500 µl binding buffer (50 mM Tris–HCl at pH 7.5, 100 mM NaCl, 0.02% Triton X-100) for 30 minutes. Subsequently, the cell lysate of TIE1-HA transfected HEK293T cells was added. After 2 hours of incubation, the beads were washed five times with Ripa buffer (50 mM Tris [pH 7.5], 1% NP-40, 0.1% SDS, 0.5% Na-deoxycholate, 150 mM NaCl) and boiled for 5 minutes at 95°C in sample buffer. For pull-down assays that include angiopoietins, TIE1-HA and ANG2-FLAG/ANG1-FLAG were co-transfected and the cell lysates were pre-cleared with Strep-TactinXT 4Flow high-capacity resin, before adding them to the Strep II tagged SVEP1 protein.

For TIE1 autophosphorylation, hdLECs were starved for 3 hours in 0.5% BSA-containing MV2 medium, followed by 1 hour of stimulation with ANG2 and SVEP1 alone or in combination. The cells were lysed in PLCLB lysis buffer (150 mM NaCl, 5% glycerol, 1% Triton X-100, 1.5 mM MgCl_2_, 2 mM CaCl_2_, 50 mM HEPES, pH 7.5, 1x Complete-EDTA-free proteinase inhibitors) followed by TIE1 immunoprecipitation using anti-TIE1 antibodies (AF619, R&D Systems) and protein G–Sepharose (GE Healthsciences Ab, #GE17-0618-01) at +4°C. After 2 hours of incubation, the beads were washed five times with PLCLB lysis buffer and boiled for 5 minutes at 95°C in sample buffer.

For analysis of pAKT, growth medium of confluent hdLECs was changed to MV2 medium containing growth supplements but lacking VEGFC. Cells were starved for 3 hours in 0.5% BSA-containing MV2 medium, followed by 40–60 minutes of stimulation with ANG2 (0.5 µg/ml) and/or SVEP1 (1 µg/ml). Cells were treated with anti-ANG2 antibody (2 µg/ml; MEDI3617 A2770, Selleckchem) for 2 hours prior to stimulation with SVEP1. The cells were lysed in lysis buffer (20 mM Tris-HCl [pH 7.4], 150 mM NaCl, 2 mM CaCl_2_, 1.5 mM MgCl_2_, 1 mM Na_3_VO_4_, 1% [v/v] Triton-X-100, 0.04% [w/v] NaN_3_, 1x Complete-EDTA-free proteinase inhibitors) or PLCLB lysis buffer and centrifuged before gel analysis.

#### Western blot

Cell lysates and cell precipitates were subjected to Western blot analysis by separation in SDS-page and blotting onto PVDF membrane. Blots were probed with primary antibodies followed by HRP coupled secondary antibodies and ECL detection (SuperSignal West Pico PLUS Chemilumineszenz-Substrat, Thermo Fisher Scientific, 34580). Blots were imaged using an Odyssey FC (LICOR). Primary and secondary antibodies used for analysis are listed in the ‘key resources table’ and are stated in the figure of the respective blot. Before using the Strep-Tactin HRP antibody, the membrane was incubated in 2,5 µg/ml Avidin (biotin blocking solution) for 10 minutes. HSC70 was used to assure equal loading of the gels, and values were normalized by the level of HSC70.

#### Immunocytochemistry and imaging

Before the treatment, hdLECs were starved for 4 hours in MV2 medium without supplements and with 0.5% FBS. After treatment with SVEP1 and/or ANG2 (623-AN R&D Systems) for 1 hour, cells were fixed with 4% PFA for 10 minutes. Afterwards, the fixed cells were permeabilized using 0,5% TritonX-100 in PBS for 5 minutes and blocked with 2% BSA for 1 hour. Incubation of the slides with the antibody was conducted overnight at 4°C (ZO-1 [Thermo Scientific, 33-9100], FOXO1 [Cell Signaling, C29H4, #2880]). After washing the slides several times with 0.1% PBS-TritonX100, the secondary antibody staining was conducted at room temperature for 50 minutes (Donkey-anti-rabbit-647 [Thermo Scientific A31573], Donkey-anti-mouse-546 [Thermo Scientific], DAPI). Slides were washed and mounted using Fluormount-G (Thermo Scientific 00-4958-02). Images were taken using ZEISS Elyra 7 super-resolution microscope in Apotome mode with 20× objective and processed with ZEN Microscopy Software (Zen blue 3.5 and Zen black). ZEISS arivis Software was used to calculate the pixel intensities inside the nucleus and in the cytoplasm. ZO1 was used to identify single cells, and the ratio was calculated for each cell.

#### Statistics and reproducibility

Data sets were tested for normality (Shapiro–Wilk) and equal variance. p-Values of data sets with normal distribution were determined by Student’s *t*-test. In case data values did not show normal distribution, a Mann–Whitney *U* test was performed instead. For multiple comparisons, the p-values were adjusted with Bonferroni correction. Results are presented as mean ± SD. Statistical tests were performed using GraphPad Prism 8 and R. For visualizations, the R package ggplot2 was used. All experiments were carried out at least three times. p-Values>0.05 were considered not significant. *p≤0.05, **p≤0.01, ***p≤0.001, ****p≤0.0001.

#### Structure prediction and modeling of SVEP1 complexes

Amino acid sequences for human SVEP1 (https://www.uniprot.org/uniprotkb/Q4LDE5), TIE1/2 (https://www.uniprot.org/uniprotkb/P35590 and https://www.uniprot.org/uniprotkb/Q02763/entry), and ANG1/2 (https://www.uniprot.org/uniprotkb/Q15389 and https://www.uniprot.org/uniprotkb/O15123/entry) were retrieved from UniProtKB. The most recent version of AlphaFold2-multimer (v2.3.2) was accessed via ColabFold (v1.6.1) (https://github.com/sokrypton/ColabFold; Mirdita and Ovchinnikov, 2026) while the DeepMind portal was used for AF3 (https://alphafoldserver.com/) modeling of >2500 amino acid jobs. For the template-free AF2.3 predictions, num recycles = auto and num models = 5 were used; models were relaxed with AMBER and ranked based on ipTM scores; in turn, default parameters were used for AF3. Top-ranked model interfaces were analyzed and visualized with PDBePISA (Krissinel and Henrick, 2007) and PyMOL v3.1.8 (The PyMOL Molecular Graphics System, Version 3.1.4.1, Schrödinger, LLC).

## Data Availability

Scripts used for data analysis available at GitHub at https://github.com/MuensterImagingNetwork/Uphoff_et_al_2025 (copy archived at Münster Imaging Network, 2026). The datasets (e.g., of images) generated during and/or analyzed during the current study are available at https://doi.org/10.6019/S-BIAD4061, any further data and source data files are included in the manuscript and supporting files. The following dataset was generated: UphoffK WendtJ Schulte-MerkerS Schulte-OstermannD 2026SVEP1 enables efficient binding of Angiopoietin-2 to the TIE1 receptor, allowing receptor phosphorylation and downstream signaling; Imaging data corresponding to Figure 3BioImage Archive10.6019/S-BIAD4061PMC1372241742960837

## Additional files

MDAR checklist

## Appendix 1

**Appendix 1—key resources table:**

Reagent type (species) or resource	Designation	Source or reference	Identifiers	Additional information
Cell line (human)	HEK293 EBNA	Manuel Koch, not authenticated		Tested negative for mycoplasma
Cell line (human)	293T HEK cells	Roukens et al., 2015, not authenticated		Tested negative for mycoplasma
Cell line (human)	Primary Human Dermal Lymphatic Endothelial Cells	PromoCell	C-12216	Tested negative for mycoplasma
Transfected construct (human)	TIE1-HA in PCEP4	Hußmann et al., 2023		TIE1 cDNA provided by Hellmut Augustin
Transfected construct (human)	TIE2-HA in PCEP4	Hußmann et al., 2023		Provided by Manuel Koch
Transfected construct (human)	ANG1-FLAG	This paper		Provided by Manuel Koch
Transfected construct (human)	ANG2-FLAG	This paper		Provided by Manuel Koch
Peptide, recombinant protein	VEGF-C (human)	R&D Systems	9199-VC	
Peptide, recombinant protein	Human recombinant protein ANG1 (human)	R&D Systems	923-AN-025	
Peptide, recombinant protein	Human recombinant protein ANG2 (human)	R&D Systems	623-AN-025	
Peptide, recombinant protein	Human ANG1-Strep II (amino acids (aa) 20–497) (human)	This paper		Provided by Manuel Koch
Peptide, recombinant protein	Human ANG2-Strep II (aa 20–495) (human)	This paper		Provided by Manuel Koch
Peptide, recombinant protein	Human C-term SVEP1-Strep II purified recombinant protein, reaching from CCP15 to the C-Term, aa 2261–3572 (human)	Hußmann et al., 2023		Provided by Manuel Koch
Peptide, recombinant protein	Human C-term SVEP1 TIE1mut- Strep II purified recombinant protein, reaching from CCP15 to the C-Term, aa 2261–3572, mutation E2568A G2569A (human)	This paper		Provided by Manuel Koch
Peptide, recombinant protein	Human complete C-term SVEP1 Strep II purified recombinant protein, reaching from EGF1 to the C-Term, aa 919 to the C-term (human)	This paper		Provided by Manuel Koch
Peptide, recombinant protein	TIE1 (ectodomain TIE1, aa: 21–760) (human)	This paper		Provided by Manuel Koch
Peptide, recombinant protein	TIE2 (ectodomain TIE2, aa: 23–745) (human)	This paper		Provided by Manuel Koch
Peptide, recombinant protein	TIE1- Strep II ALFA-tag (ectodomain TIE1, aa: 21–760) (human)	This paper		Provided by Manuel Koch
Peptide, recombinant protein	SVEP1-Strep II CCP domain 19_20_21 Purified recombinant protein (aa 2496–2714) (human)	This paper		Provided by Manuel Koch
Peptide, recombinant protein	SVEP1-Strep II CCP domain 19_20 Purified recombinant protein (aa 2496–2610) (human)	This paper		Provided by Manuel Koch
Peptide, recombinant protein	SVEP1-Strep II domain 19_20mut_21 Purified recombinant protein (aa 2496–2714, E2568A G2569A) (human)	This paper		Provided by Manuel Koch
Peptide, recombinant protein	VEGFC-Strep II Purified recombinant protein (human)	This paper		Provided by Manuel Koch
Antibody	Anti-HA (rat, monoclonal)	Roche	11867423001	1:10,000
Antibody	Anti-FLAG (rabbit, polyclonal)	Sigma-Aldrich	F1804	1:10,000
Antibody	Strep-Tactin HRP conjugate	Iba-lifesciences	2-1502-001	1:10,000
Antibody	Anti-goat (donkey, polyclonal) 488	Thermo Scientific	A11055	1:500
Antibody	Anti-mouse (donkey, polyclonal) 546	Thermo Scientific	A-21123	1:500
Antibody	Anti-rabbit (donkey, polyclonal) 647	Thermo Scientific	A31573	1:500
Antibody	Anti-mouse (donkey, polyclonal) HRP	Jackson ImmunoResearch	715-035-150	1:15,000
Antibody	Anti-goat (donkey, polyclonal) HRP	Immuno Reagents Inc.	DkxGt-003-EHRPX	1:15,000
Antibody	Anti-rabbit (donkey polyclonal) HRP	Jackson ImmunoResearch	711-035-152	1:15,000
Antibody	Anti-TIE2 (goat, polyclonal)	R&D Systems	AF313	1:300
Antibody	Anti-TIE1 (goat, polyclonal)	R&D Systems	AF619	1:300
Antibody	Anti-phospho-TIE2 (rabbit, polyclonal)	R&D Systems	pY992, AF2720	1:300
Antibody	Anti-phosphotyrosine (mouse, monoclonal)	Millipore	05–321, clone 4G10,	1:500
Antibody	Phospho-AKT (S473) (rabbit, polyclonal)	Cell Signaling Technology	9271	1:2000
Antibody	Anti-total AKT (mouse, monoclonal) anti-total AKT (rabbit, polyclonal)	Cell Signaling Technology	2920, 9272	1:1000
Antibody	Phospho-ERK	Cell Signaling Technology	43705	1:2000
Antibody	Total ERK (mouse, monoclonal)	Cell Signaling Technology	4696	1:4000
Antibody	ZO-1 (mouse, monoclonal)	Thermo Scientific	33–9100	1:300
Antibody	FOXO1 (rabbit, monoclonal)	Cell Signaling	C29H4, 2880	1:300
Antibody	HSC70 (mouse, monoclonal)	Sigma-Aldrich	MABE1120	1:1000
Antibody	Anti-ANGPT2 (MEDI3617)	Selleckchem	A2770	2 µg/ml
Chemical compound, drug	PierceAvidin	Thermo Scientific	21121	
Chemical compound, drug	Endothelial Cell Growth Medium MV2	PromoCell	C-22022	
Chemical compound, drug	Endothelial Cell Growth Medium MV2	PromoCell	C-39226	
Chemical compound, drug	FCS	Merck Chemicals GmbH	F7524	
Chemical compound, drug	DMEM/F-12, GlutaMAX Supplement	Thermo Fisher Scientific	10565018	
Chemical compound, drug	Fibronectin (1 ug/ml)	Sigma	F1141-1MG	
Chemical compound, drug	SuperSignal West Pico PLUS Chemilumineszenz-Substrat	Thermo Fisher Scientific	34580	
Chemical compound, drug	Fluormount-G	Thermo Scientific	00-4958-02	
Chemical compound, drug	Strep-Tactin Superflow high capacity resin	IBA Lifesciences GmbH	2-1208-002	
Chemical compound, drug	Lipofectamin 2000 transfection reagent	Thermo Fisher Scientific	11668030	
Chemical compound, drug	1-phenyl-2-thiourea (75 mM)	Sigma	P7629	
Chemical compound, drug	Tricaine	Sigma	A5040	
Chemical compound, drug	0.8% low melting agarose	Thermo Fisher	16520100	
Chemical compound, drug	Protein G–Sepharose	GE Healthsciences	GE17-0618-01	
Chemical compound, drug	PrimeSTAR Max DNA Polymerase	TaKaRa	R045A	
Commercial assay or kit	QIAquick PCR purification kit	QIAGEN	28106	
Commercial assay or kit	GeneJET gel extraction kit	Thermo Scientific	K0503	
Chemical compound, drug	DpnI,	Promega	R623A	
Chemical compound, drug	AvrII	New England Biolabs	R0174S	
Chemical compound, drug	T4 DNA Ligase (1–3 u/µl)	Promega	M180A	
Chemical compound, drug	T7 RiboMAX TM Large Scale RNA Production System	Promega	#P1280	
Chemical compound, drug	Ribo m7G Cap Analog	Promega	#P1711	
Chemical compound, drug	2× rapid ligation buffer	Promega	#C671A	
Chemical compound, drug	10× ligation buffer	Promega	#C126B	
Cell line (*Escherichia coli*)	Promega JM109 Competent *E. coli*	Promega	#L1001	
Cell line (*E. coli*)	NEB 5-alpha Competent *E. coli*	Promega	#C2987	
Software, algorithm	RStudio	2023.06.0 Build 421		
Software, algorithm	Fiji-ImageJ	Version 1.54f		
Software, algorithm	ZEISS Elyra 7 superresolution microscope			
Software, algorithm	ZEN Microscopy Software	Zen blue and Zen black		
Software, algorithm	Leica	LAS X 3.5.7.23225		
Software, algorithm	Adobe Illustrator	2023		
Software, algorithm	Arivis	Vision 4D 4.1		
Software, algorithm	AlphaFold2-multimer (AF2.3.2) (Evans et al., 2022)	Accessed via ColabFold 1.5.5 (https://github.com/sokrypton/ColabFold),		
Software, algorithm	DeepMind portal	https://alphafoldserver.com/		
Software, algorithm	Predictome web site	https://predictomes.org/tools/af3/		
Software, algorithm	PyMOL3.04	http://www.pymol.org		
Sequence-based reagent	TIE1 siRNA	IDT	234070784	hs.Ri.TIE1.13
Sequence-based reagent	TEK siRNA	Thermo Fisher Scientific	Catalog #4390824, Catalog #4392420,	siRNA ID #s13984, siRNA ID #s224717
Other	Immobilon-P Membrane, PVDF	Millipore Thermo	IPVH00010	
