# Supplementary material for: SVEP1 enables efficient binding of angiopoietin-2 to the TIE1 receptor, allowing receptor phosphorylation and downstream signaling
Source: eLife. 2026 Oct 30;14:RP106338. doi: 10.7554/eLife.106338 (PMC13722417; doi:10.7554/eLife.106338)
Supplement: Supplementary file 7 [file elife-106338-fig1-figsupp3-data2.zip › Figure 1-figure supplement 3 Raw Data.pdf]

RAW data Figure Figure 1-figure supplement 3

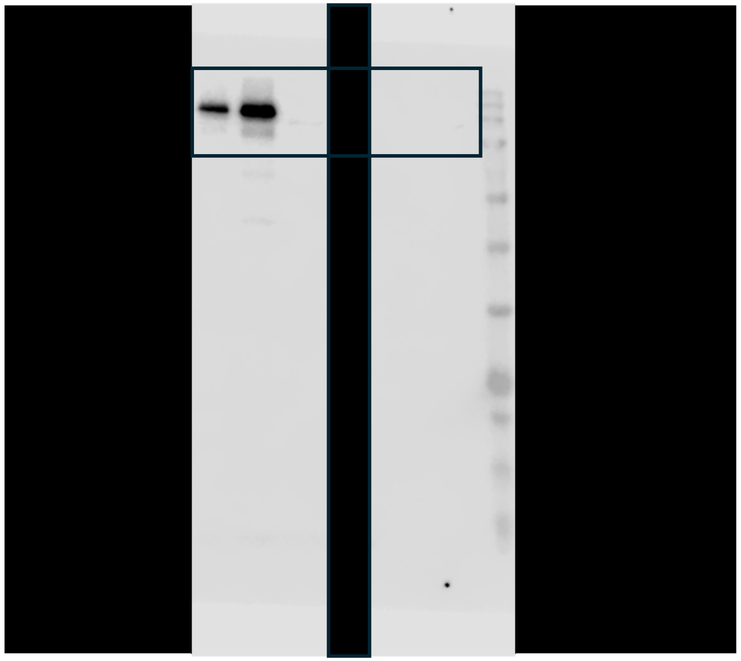

IP12(1)_antiHA_5sec

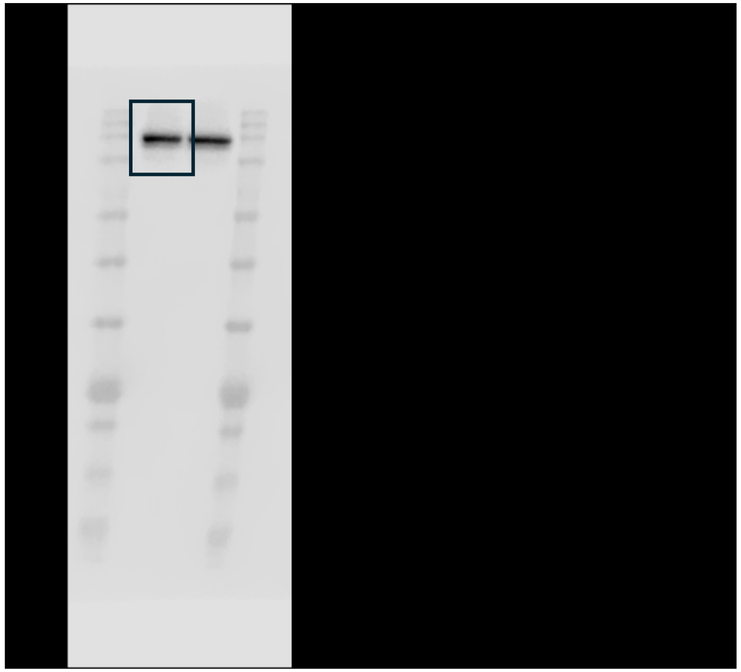

IP12(2)_totallysate_antiHA_5sec

RAW data Figure Figure 1-figure supplement 3

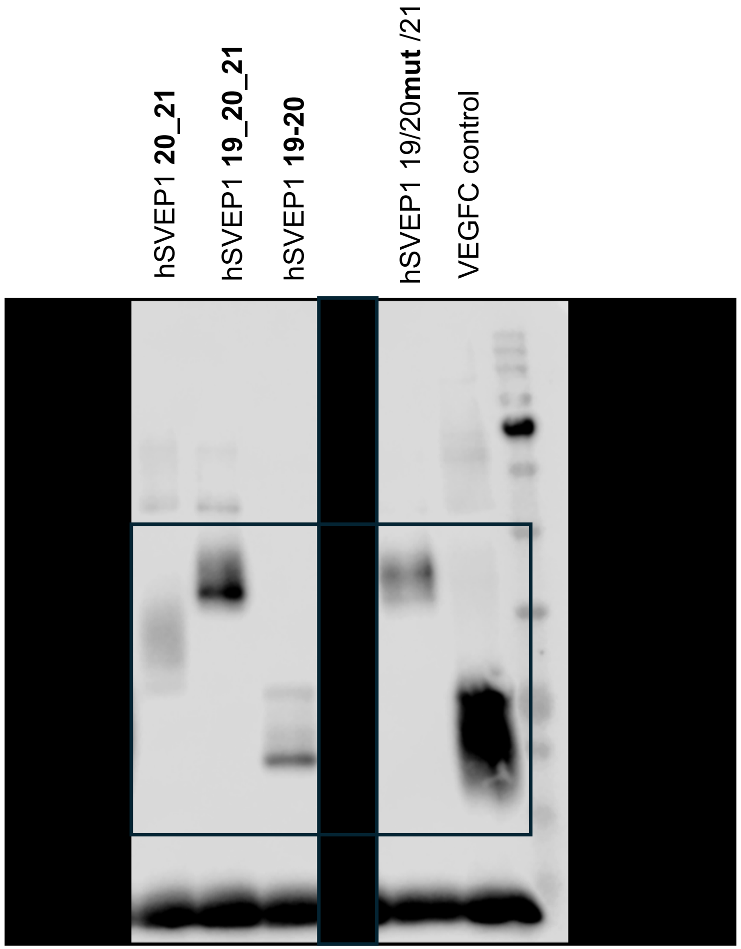

IP12(2)_antiStrepTac_5sec
