# Supplementary material for: SVEP1 enables efficient binding of angiopoietin-2 to the TIE1 receptor, allowing receptor phosphorylation and downstream signaling
Source: eLife. 2026 Oct 30;14:RP106338. doi: 10.7554/eLife.106338 (PMC13722417; doi:10.7554/eLife.106338)
Supplement: Supplementary file 9 [file elife-106338-fig2-data2.zip › Figure2 Raw Data.pdf]

RAW data Figure 3b and c

S, S+A1, S+A2

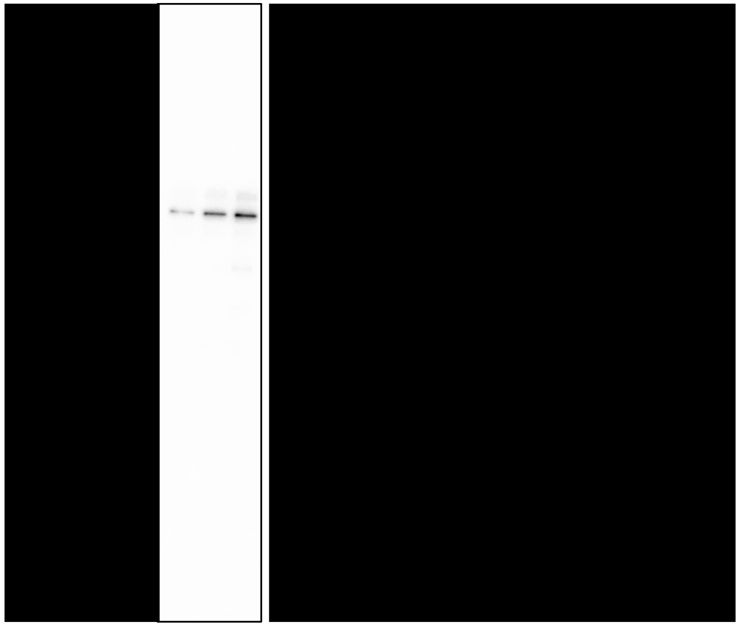

IP6_antiHA_5sec

S, S+A1, S+A2

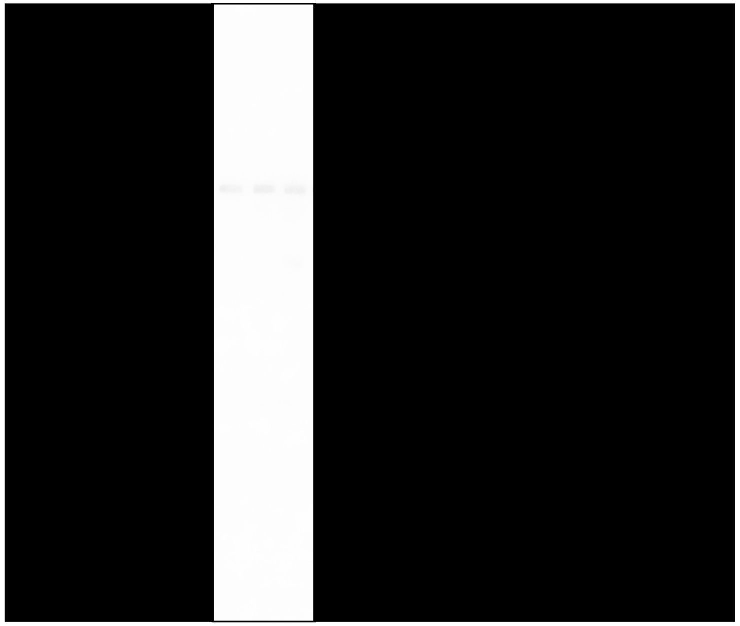

IP6_antiStrepTac_5sec

RAW data Figure 3b and c

S, S+A1, S+A2

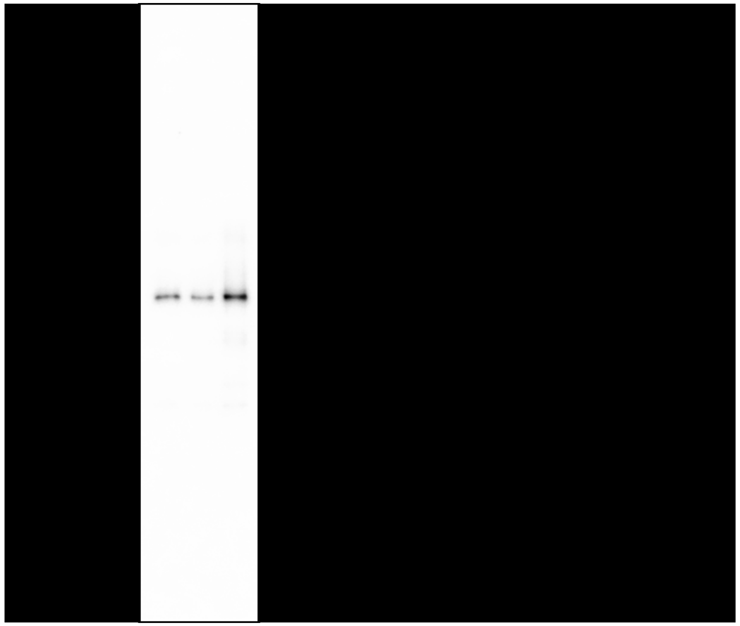

IP8_antiHA_5sec

S, S+A1, S+A2

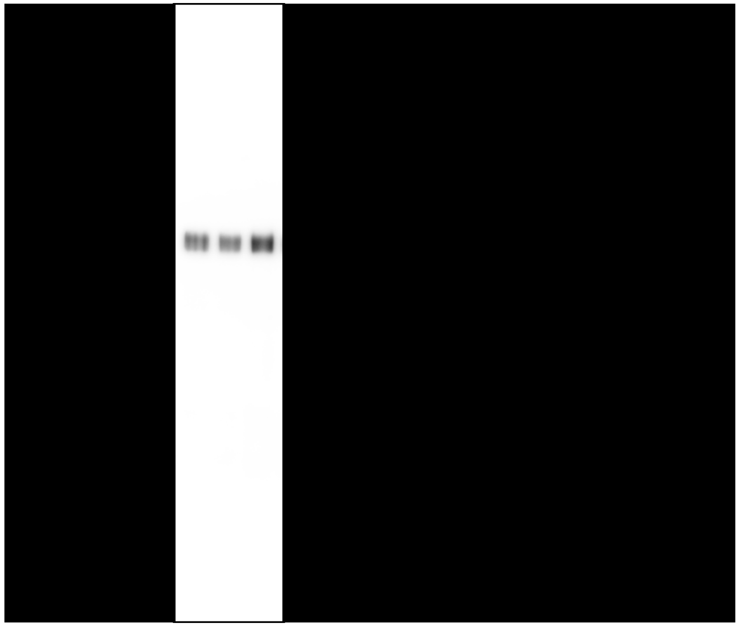

IP8_antiStrepTac_5sec

RAW data Figure 3b and c

S, S+A1, S+A2, S, S+A1, S+A2

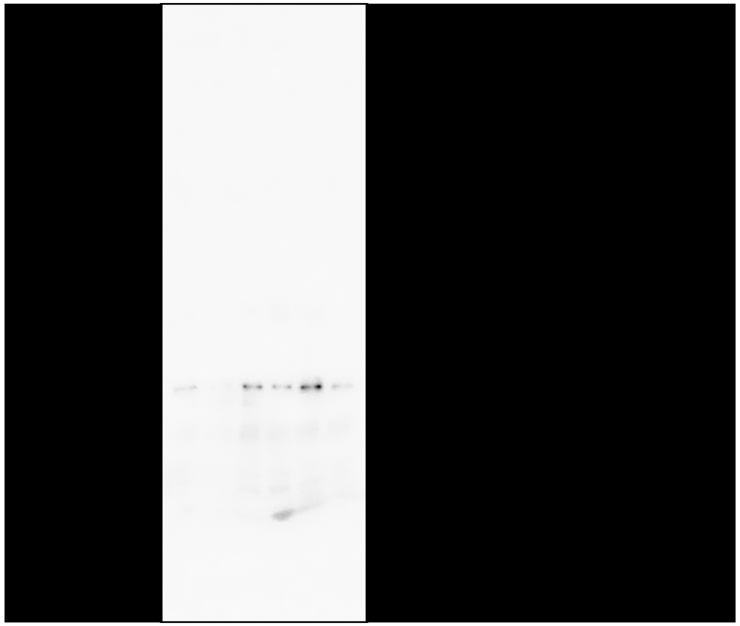

IP10(1)_antiHA_5sec

S, S+A1, S+A2, S, S+A1, S+A2

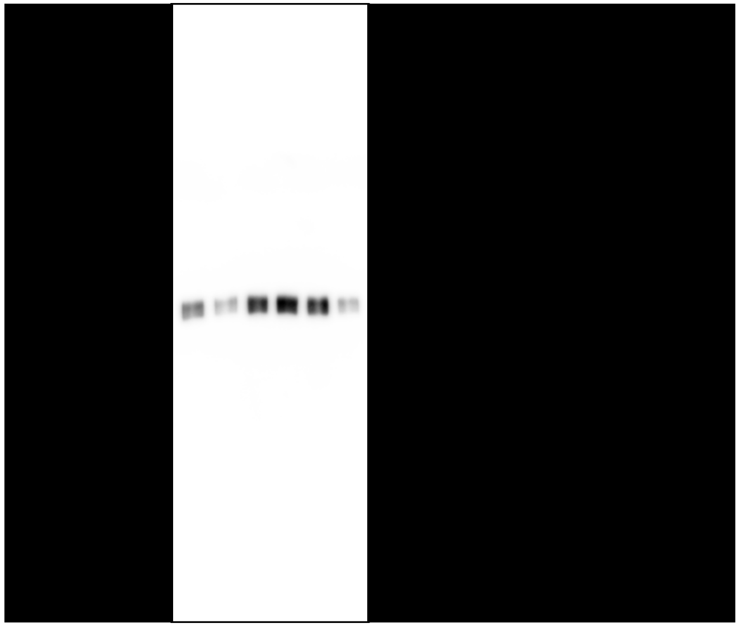

IP10(1)_antiStrepTac_5sec

RAW data Figure 3b and c

S, S+A1, S+A2, S, S+A1, S+A2

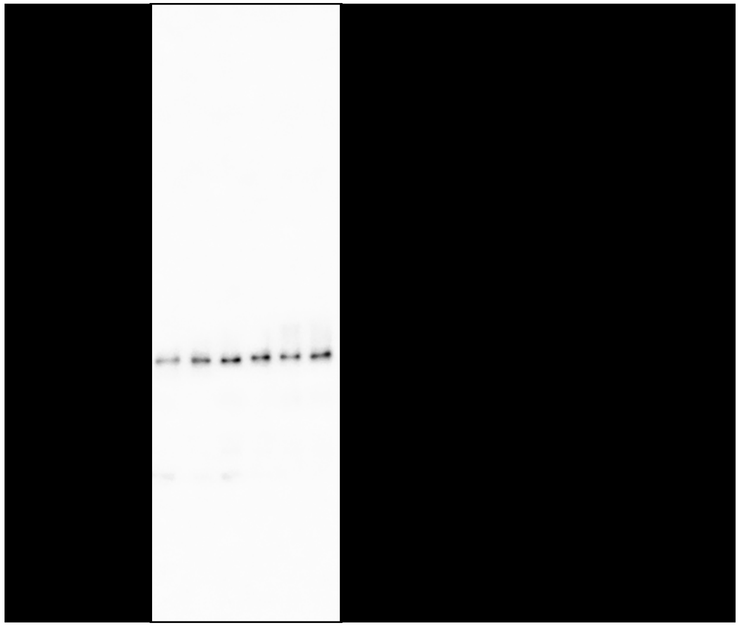

IP10(2)_antiHA_5sec

S, S+A1, S+A2, S, S+A1, S+A2

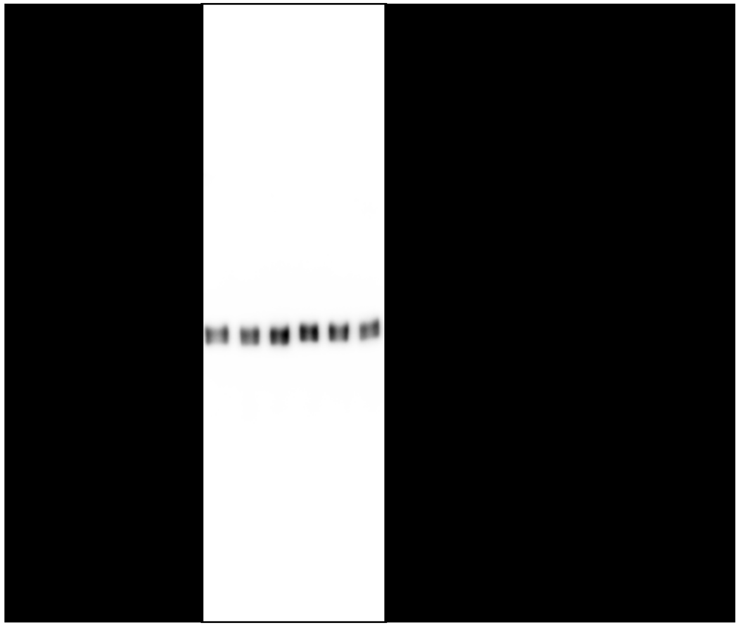

IP10(2)_antiStrepTac_5sec

```
.y.        group1          group2          n1    n2 statistic     p p.adj p.adj.signif
<chr>      <chr>           <chr>         <int> <int>    <dbl> <dbl> <dbl> <chr>
normalized TIE1_SVEP1      TIE1_SVEP1_ANG1     6     6       18 1     1     ns
normalized TIE1_SVEP1      TIE1_SVEP1_ANG2     6     6        0 0.003 0.008 **
normalized TIE1_SVEP1_ANG1 TIE1_SVEP1_ANG2     6     6       14 0.589 1     ns
```

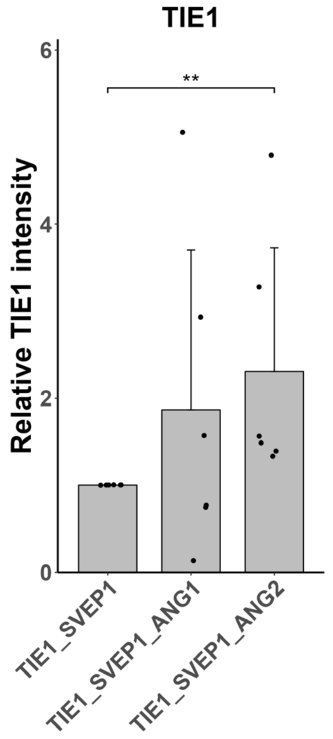

	Condition	antiHA	antiStrepTac	HA/StrepT	normalized
IP6	TIE1_SVEP1	1.917	10.819	0,17720281	1
IP6	TIE1_SVEP1_ANG1	6.125	11.815	0,51843578	2,92566336
IP6	TIE1_SVEP1_ANG2	8.966	10.571	0,84817833	4,78648343
IP8	TIE1_SVEP1	5.483	15.648	0,35040502	1
IP8	TIE1_SVEP1_ANG1	3.432	13.173	0,26048823	0,74339184
IP8	TIE1_SVEP1_ANG2	11.220	20.495	0,54747582	1,56240863
IP10 (2)	TIE1_SVEP1	4.879	16.944	0,28796158	1
IP10 (2)	TIE1_SVEP1_ANG1	7.538	16.657	0,45254423	1,5715438
IP10 (2)	TIE1_SVEP1_ANG2	8.831	22.000	0,40143822	1,39406868
IP10 (2)	TIE1_SVEP1	8.963	22.053	0,4064281	1
IP10 (2)	TIE1_SVEP1_ANG1	6.102	19.462	0,31352875	0,77142488
IP10 (2)	TIE1_SVEP1_ANG2	9.038	16.629	0,54352531	1,33732217
IP10 (1)	TIE1_SVEP1	1.366	10.924	0,12507572	1
IP10 (1)	TIE1_SVEP1_ANG1	88	5.336	0,01640382	0,13115114
IP10 (1)	TIE1_SVEP1_ANG2	3.257	17.499	0,18610428	1,48793291
IP10 (1)	TIE1_SVEP1	2.010	21.519	0,09341163	1
IP10 (1)	TIE1_SVEP1_ANG1	8.622	18.240	0,47267817	5,0601638
IP10 (1)	TIE1_SVEP1_ANG2	1.584	5.167	0,30655927	3,28181037
