# Supplementary material for: SVEP1 enables efficient binding of angiopoietin-2 to the TIE1 receptor, allowing receptor phosphorylation and downstream signaling
Source: eLife. 2026 Oct 30;14:RP106338. doi: 10.7554/eLife.106338 (PMC13722417; doi:10.7554/eLife.106338)

Figure 4B Raw Data

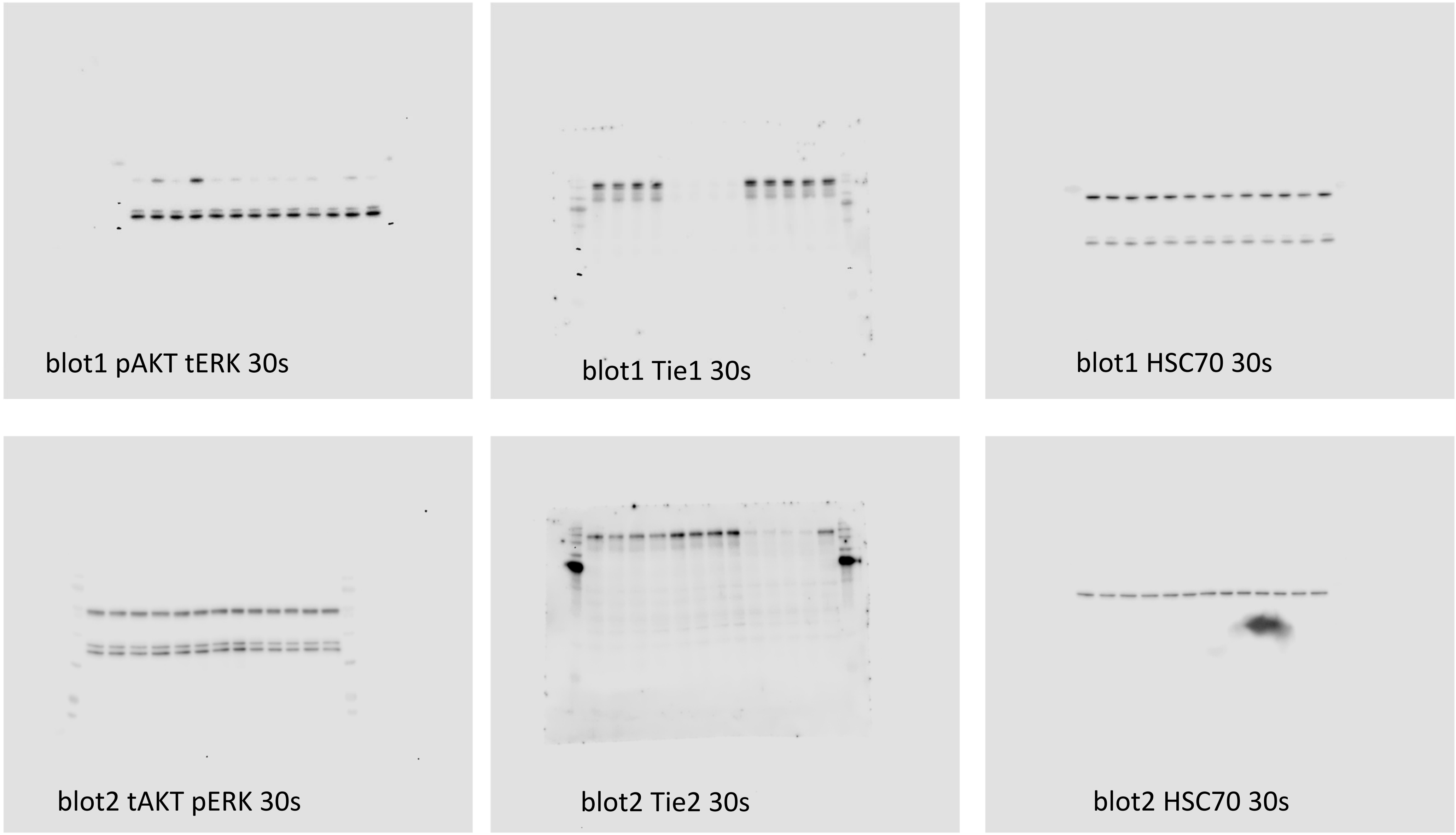

Supplement: Supplementary file 13 [file elife-106338-fig3-data2.zip › Figure 3 - source data 2/Figure 3B Raw Data.pdf]
