# Supplementary material for: SVEP1 enables efficient binding of angiopoietin-2 to the TIE1 receptor, allowing receptor phosphorylation and downstream signaling
Source: eLife. 2026 Oct 30;14:RP106338. doi: 10.7554/eLife.106338 (PMC13722417; doi:10.7554/eLife.106338)

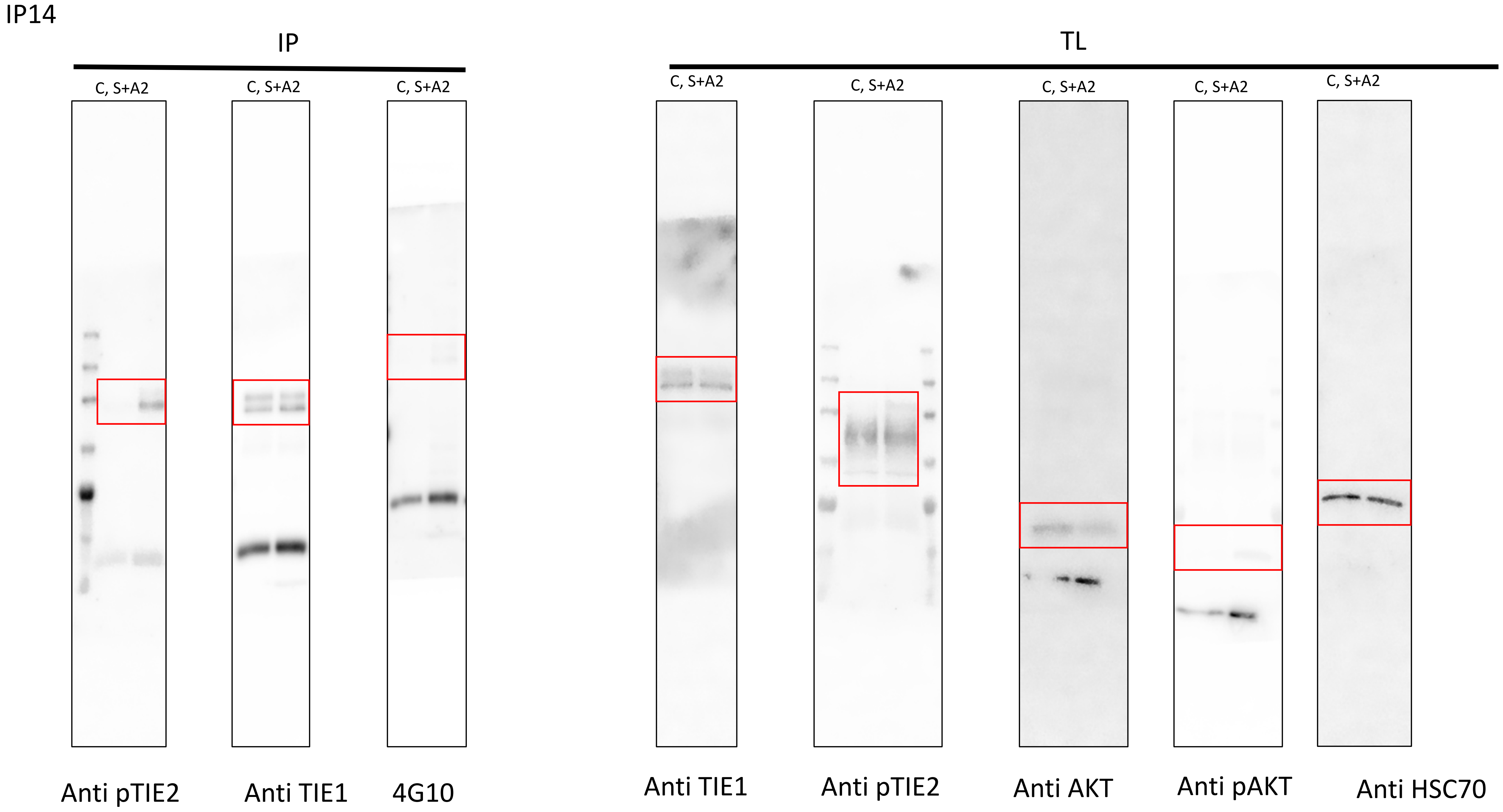
IP14
IP
TL
C, S+A2
C, S+A2
C, S+A2
C, S+A2
C, S+A2
C, S+A2
C, S+A2
C, S+A2
Anti pTIE2
Anti TIE1
4G10
Anti TIE1
Anti pTIE2
Anti AKT
Anti pAKT
Anti HSC70

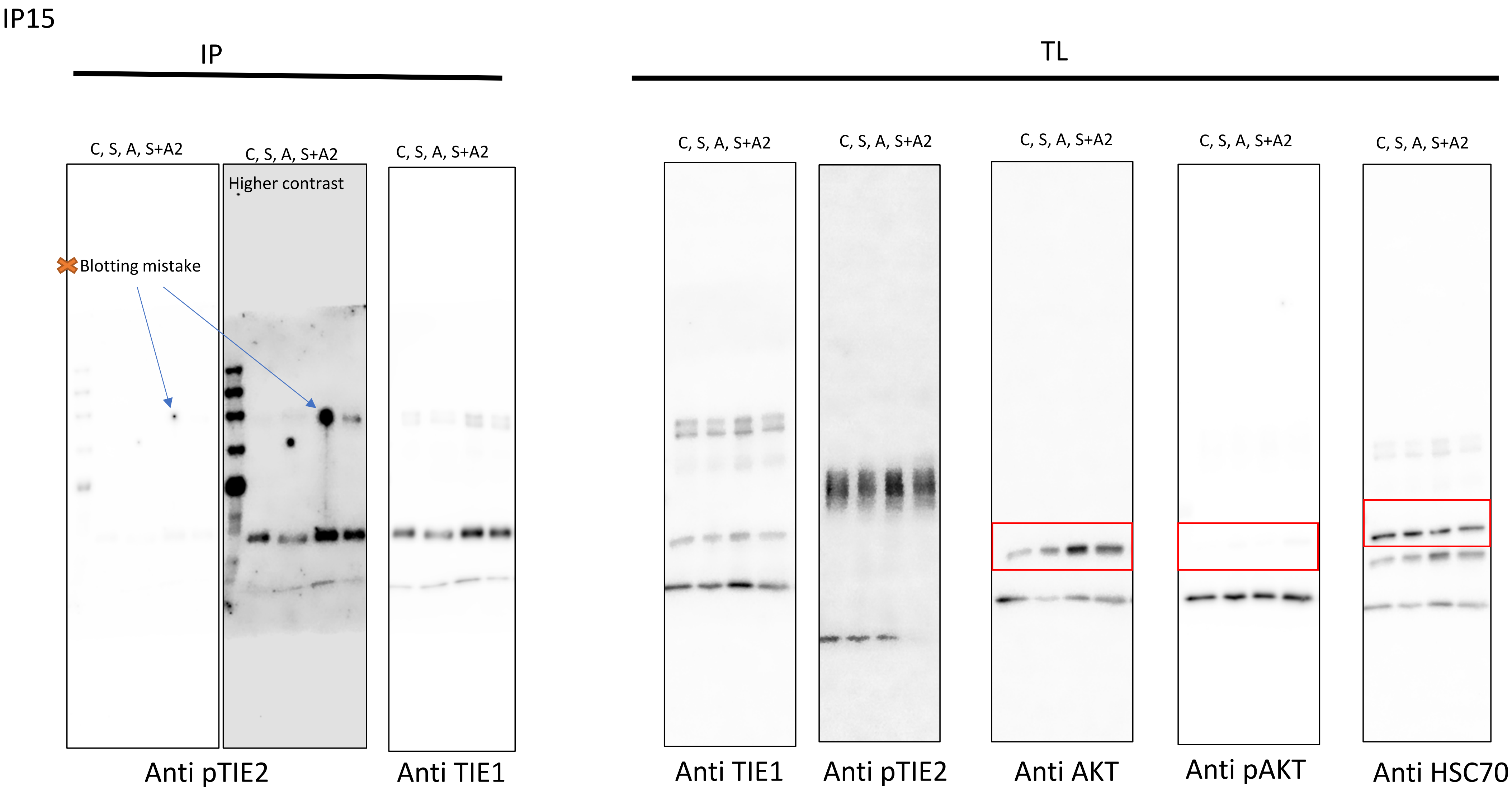
IP15
IP
TL
C, S, A, S+A2
C, S, A, S+A2
C, S, A, S+A2
Higher contrast
Blotting mistake
Anti pTIE2
Anti TIE1
C, S, A, S+A2
C, S, A, S+A2
C, S, A, S+A2
C, S, A, S+A2
C, S, A, S+A2
Anti TIE1
Anti pTIE2
Anti AKT
Anti pAKT
Anti HSC70

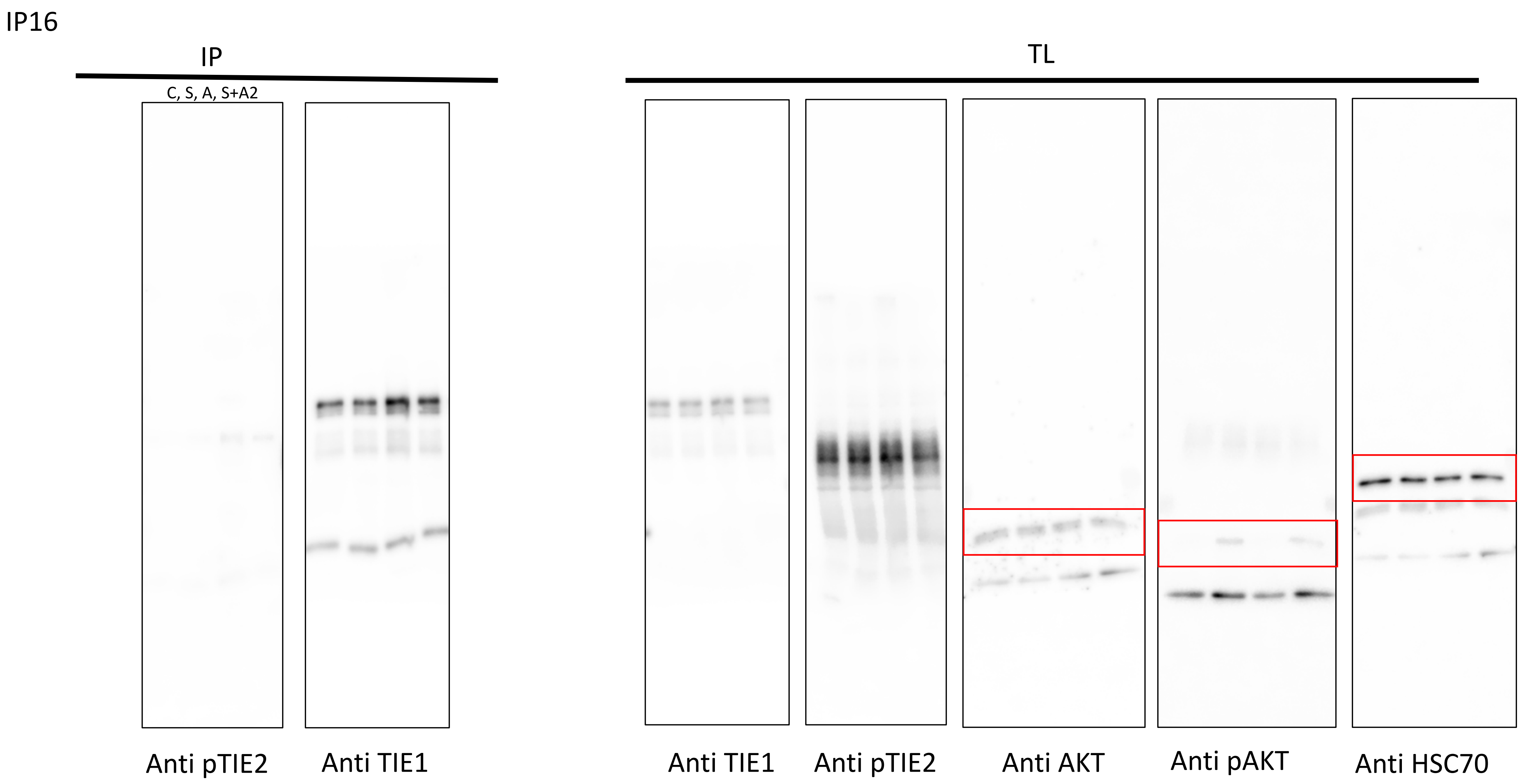
IP16
IP
C, S, A, S+A2
TL
Anti pTIE2
Anti TIE1
Anti TIE1
Anti pTIE2
Anti AKT
Anti pAKT
Anti HSC70

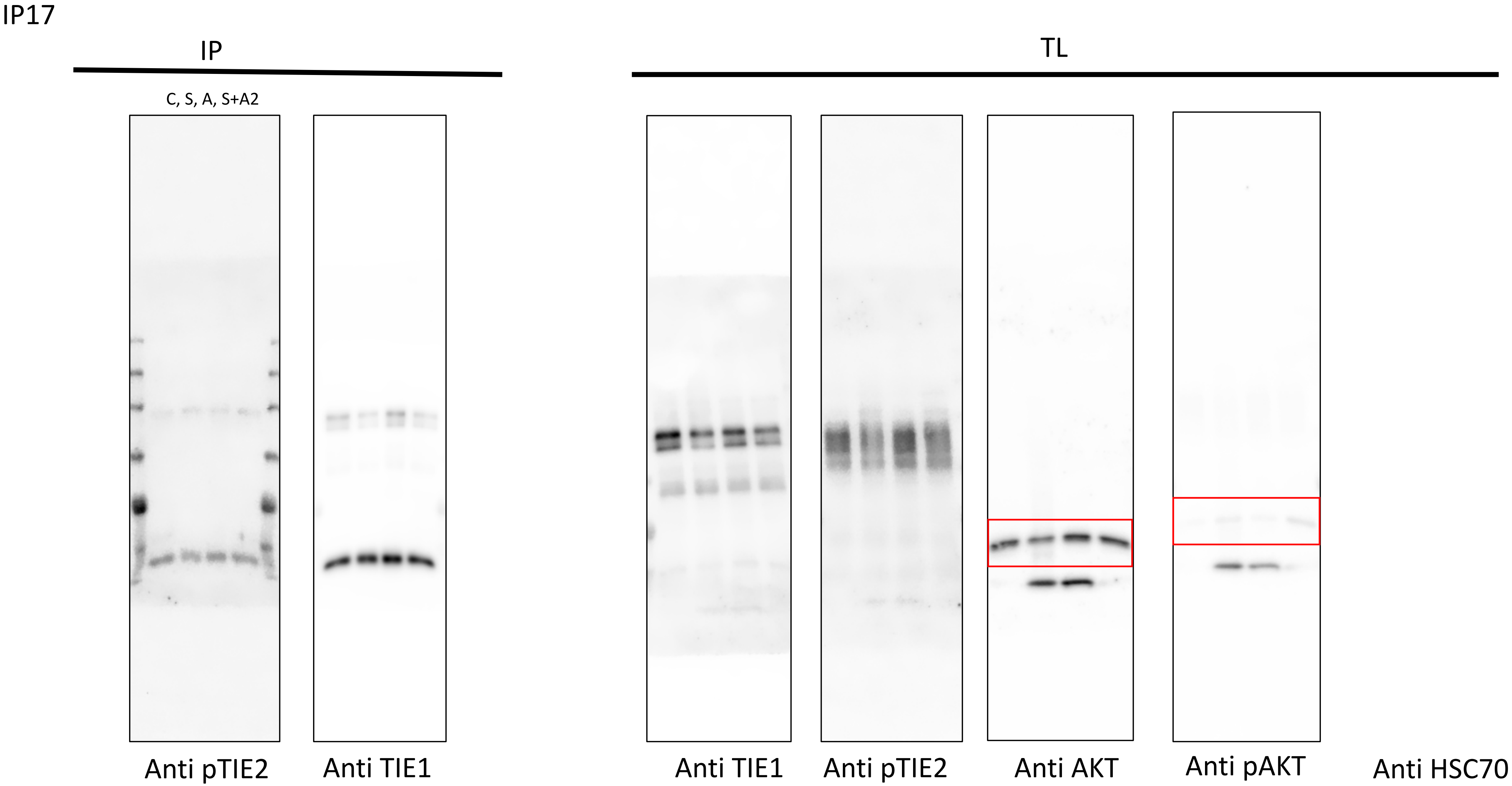
IP17
IP
TL
C, S, A, S+A2
Anti pTIE2
Anti TIE1
Anti TIE1
Anti pTIE2
Anti AKT
Anti pAKT
Anti HSC70

IP18

IP

TL

C, S, A, S+A2

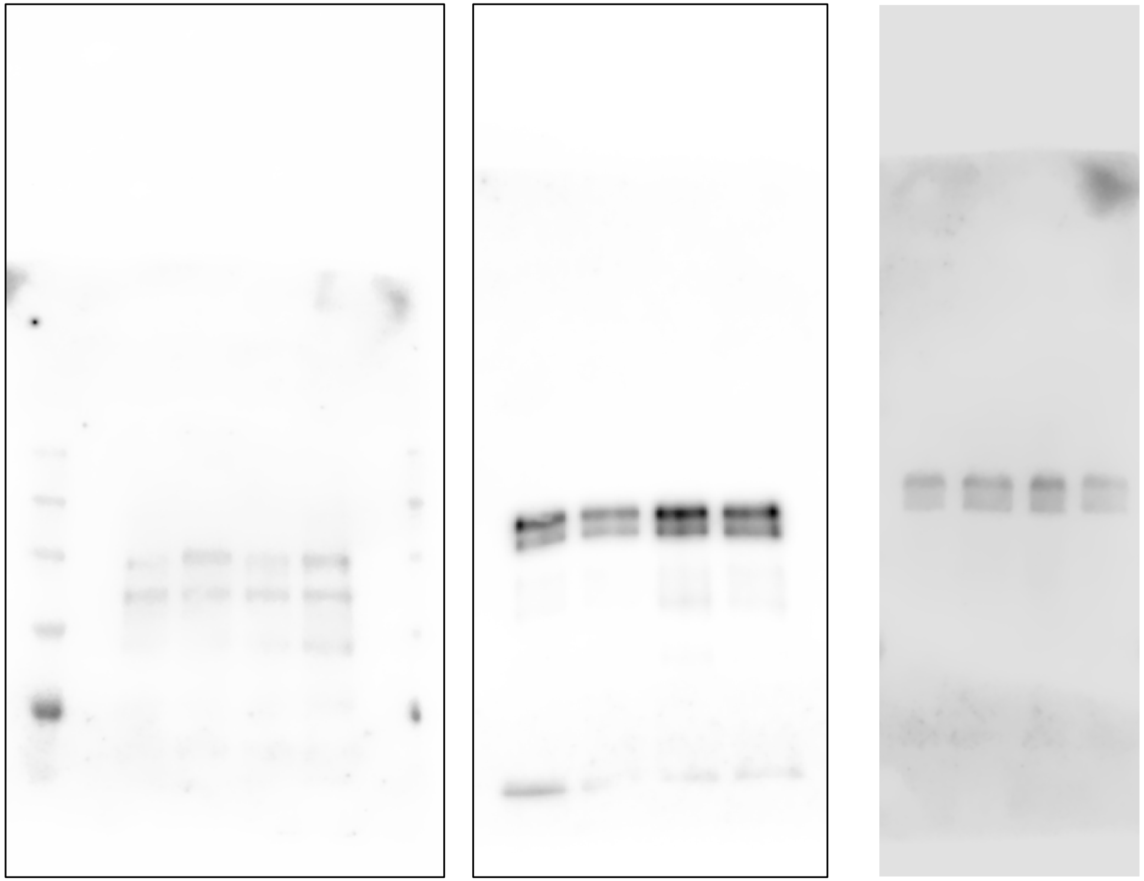

Anti pTIE2 Anti TIE1 Anti TIE1 Anti pTIE2 Anti AKT Anti pAKT Anti HSC70

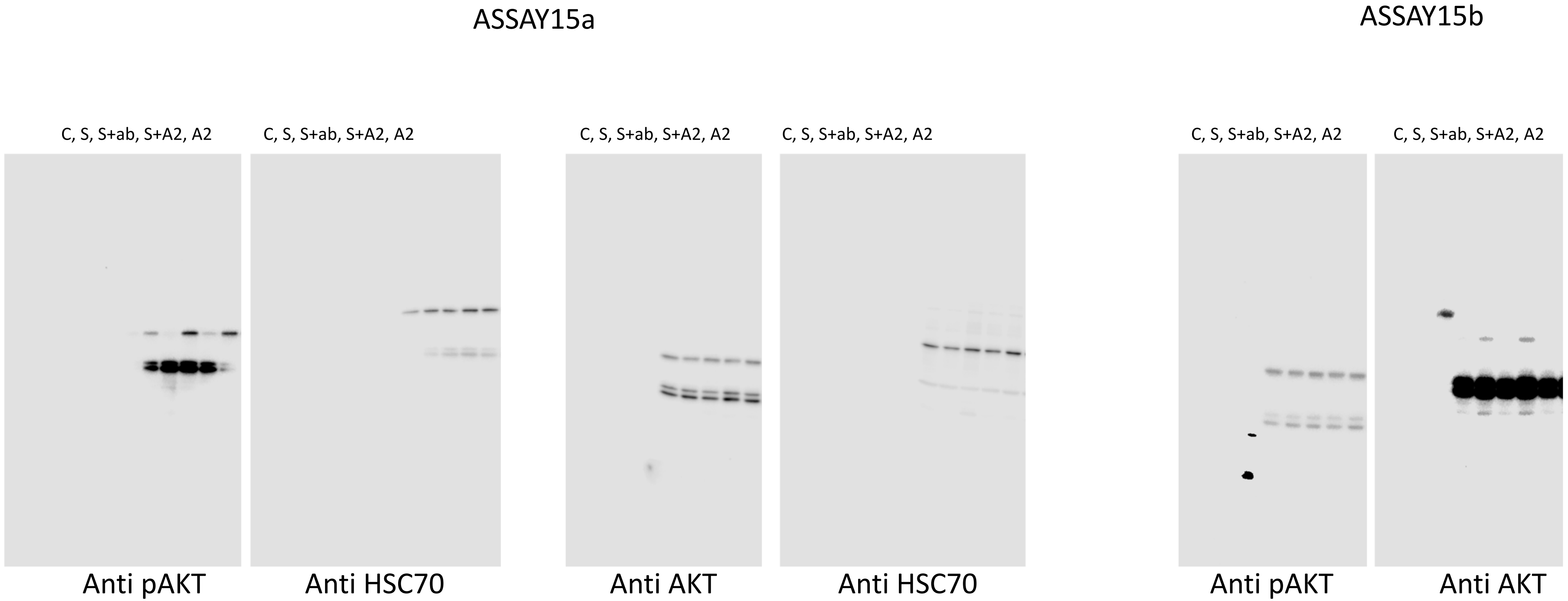

ASSAY15a
ASSAY15b
C, S, S+ab, S+A2, A2
C, S, S+ab, S+A2, A2
C, S, S+ab, S+A2, A2
C, S, S+ab, S+A2, A2
C, S, S+ab, S+A2, A2
C, S, S+ab, S+A2, A2
Anti pAKT
Anti HSC70
Anti AKT
Anti HSC70
Anti pAKT
Anti AKT

ASSAY16a

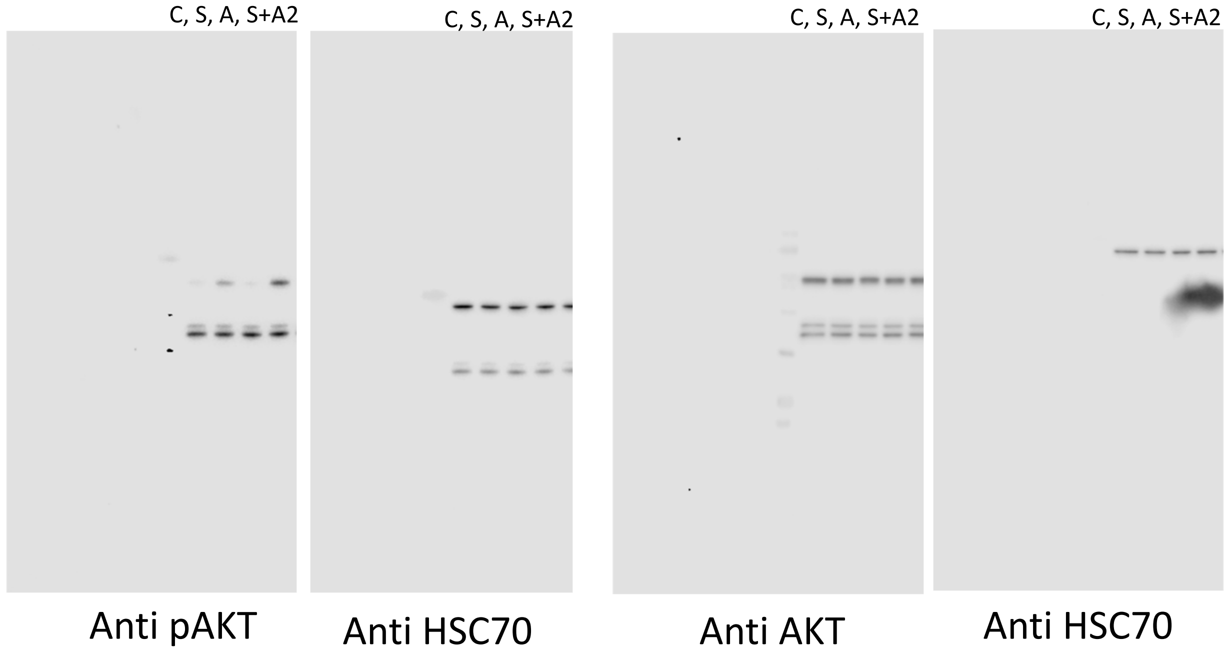

ASSAY16b

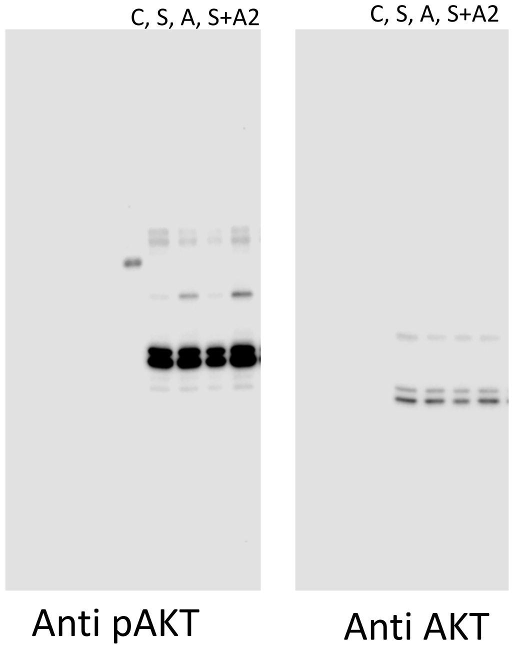

ASSAY18

C, S, A, S+A2

C, S, A, S+A2

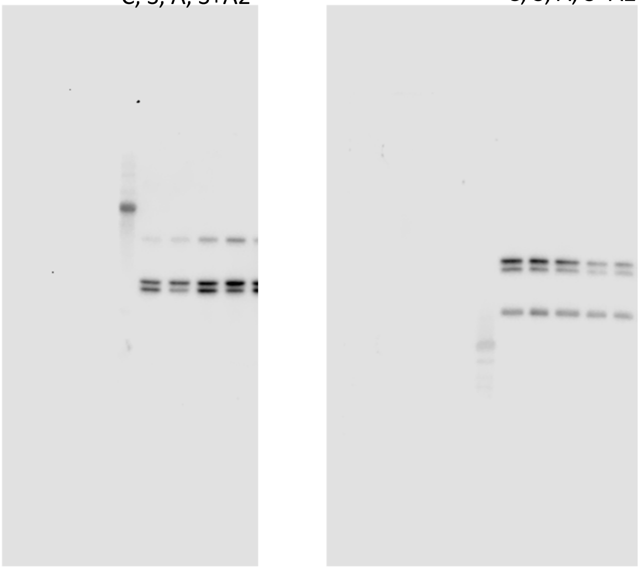

Anti pAKT

Anti AKT

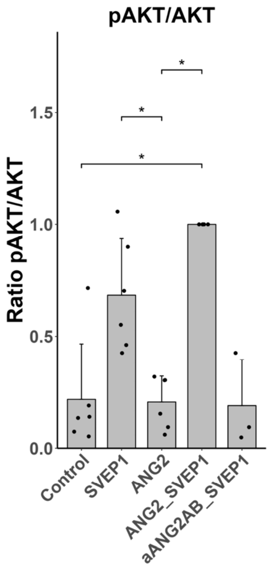

Assay	Condition	pAKT_AKT
a15	Control	1
a15	SVEP1	2,79270343
a15	ANG2	0,81049664
a15	ANG2_SVEP1	4,66536037
a15	aANG2AB_SVEP1	0,59799407
a16	Control	1
a16	SVEP1	6,75894834
a16	ANG2	1,42379628
a16	ANG2_SVEP1	14,1687481
a16	aANG2AB_SVEP1	0,85082997
a18b	Control	1
a18b	SVEP1	2,90320264
a18b	ANG2	1,59420237
a18b	ANG2_SVEP1	5,26072679
a18b	aANG2AB_SVEP1	1,27237453
IP14	Control	1
IP14	ANG2_SVEP1	12,4668385
IP15	Control	1
IP15	SVEP1	1,47943651
IP15	ANG2	0,44698766
IP15	ANG2_SVEP1	1,39970323
IP16	Control	1
IP16	SVEP1	16,9179235
IP16	ANG2	1,14592808
IP16	ANG2_SVEP1	18,8318076
IP17	Control	1
IP17	SVEP1	5,02364327
IP17	ANG2	2,17504945
IP17	ANG2_SVEP1	7,15679373

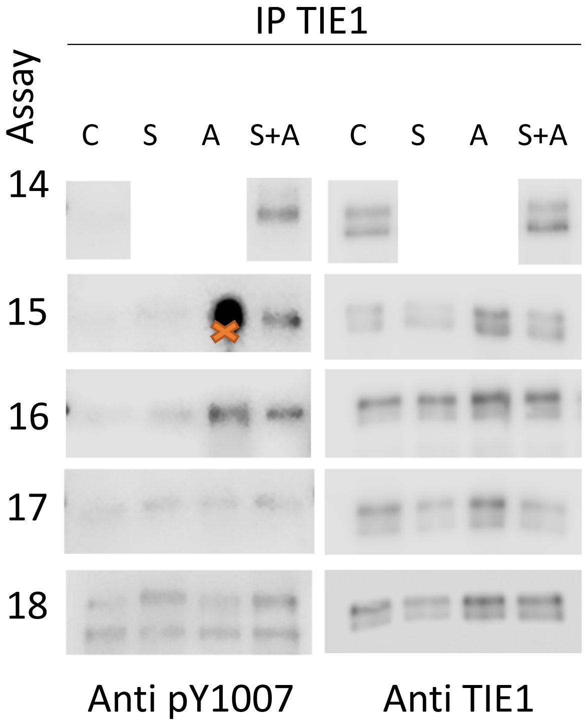

group1	group2	n1	n2	p	p.adj	p.adj.signif
Control	SVEP1	5	4	0,011	0,065	ns
Control	ANG2	5	3	0,495	1	ns
Control	SVEP1_ANG2	5	5	0,007	0,045	*
SVEP1	ANG2	4	3	0,114	0,684	ns
SVEP1	SVEP1_ANG2	4	5	0,905	1	ns
ANG2	SVEP1_ANG2	3	5	0,143	0,858	ns

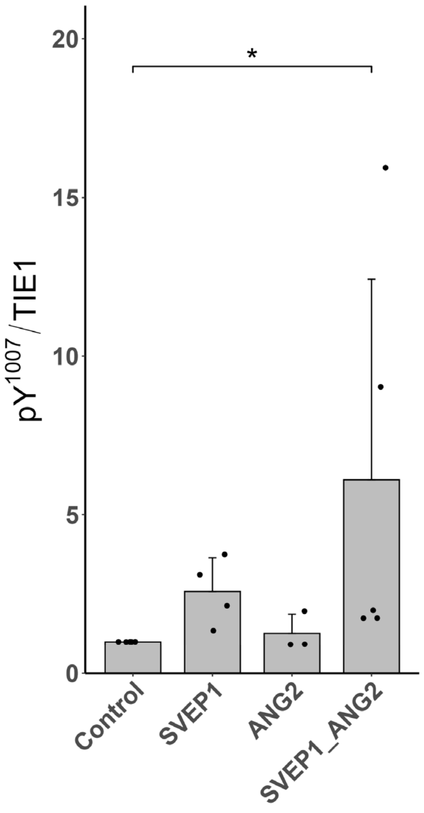

Assay 14-18

group1	group2	n1	n2	p	p.adj	p.adj.signif
Control	SVEP1	3	3	0,078	0,466	ns
Control	ANG2	3	3	0,485	1	ns
Control	SVEP1_ANG2	3	3	0,000534	0,003	**
SVEP1	ANG2	3	3	0,206	1	ns
SVEP1	SVEP1_ANG2	3	3	0,513	1	ns
ANG2	SVEP1_ANG2	3	3	0,192	1	ns

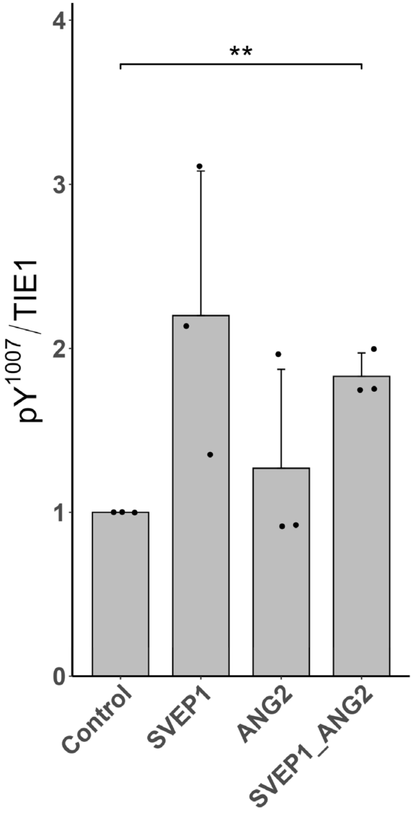

Assay 16-18

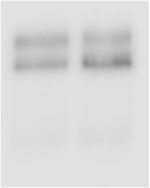

Assay

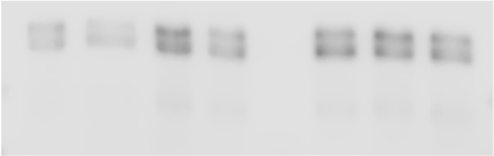

14

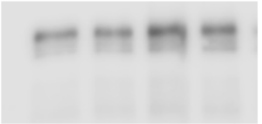

15

16

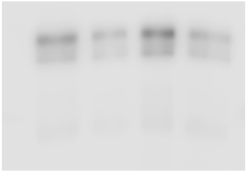

17

18

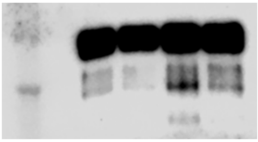

Supplement: Supplementary file 13 [file elife-106338-fig3-data2.zip › Figure 3 - source data 2/Figure3A-D-E Raw Data.pdf]
