# Supplementary material for: SVEP1 enables efficient binding of angiopoietin-2 to the TIE1 receptor, allowing receptor phosphorylation and downstream signaling
Source: eLife. 2026 Oct 30;14:RP106338. doi: 10.7554/eLife.106338 (PMC13722417; doi:10.7554/eLife.106338)
Supplement: Peer review history

# Editor report: eLife Assessment

**DOI:** 10.7554/eLife.106338.3.sa0

**Authors:** Volker Dötsch

**Affiliations:** Goethe University Frankfurt Germany

**Keywords:** Important, Solid

This article focuses on developing a structural model of how the multidomain ECM protein SVEP1 enables angiopoietin (ANG) binding to the orphan receptor TIE1, resulting in downstream receptor phosphorylation and signaling. This is an **important** study, based on **solid** data. The results will be of interest to scientists working in vascular biology and RTK signaling.

# Referee report: Reviewer #1 (Public review)

**DOI:** 10.7554/eLife.106338.3.sa1

Summary:

In this manuscript, Uphoff et al. propose a structural and mechanistic model in which the multidomain ECM protein SVEP1 enables Angiopoietin (ANG) binding to the orphan receptor TIE1, thereby promoting downstream receptor phosphorylation and signaling. Using AlphaFold-based modeling, the authors predict that the CCP20 domain of SVEP1 binds to TIE1, creating a composite surface that facilitates Angiopoietin association and TIE1 activation. The resulting ternary model (SVEP1-TIE1-ANG) offers a structural rationale for how SVEP1 converts TIE1 into a functional, ligand-responsive receptor. Additional models and biological assays suggest roles for other domains of SVEP1, such as CCP5-EGF-L7, although these interactions are predicted with low confidence. The authors interpret these findings as the first structural framework for how SVEP1 enables ANG-TIE1 signaling.

Strengths:

(1) The central hypothesis - that SVEP1 enables ANG binding to the orphan receptor TIE1 - is biologically compelling and addresses an important question in vascular biology.

(2) The AlphaFold-predicted ternary complex (SVEP1-TIE1-ANG) is plausible, high-confidence, and structurally consistent with prior functional data (e.g., poly-Ala scanning from Sato-Nishiuchi et al.).

(3) The authors' model offers a potential explanation for the previously observed role of SVEP1 in enhancing ANG signaling through TIE1 and may represent the first structural insight into TIE1's transition from orphan to ligand-activated receptor.

(4) The potential clinical implication - that a combinatorial ligand (ANG+SVEP1) can activate TIE1- could have translational relevance for vascular leak and inflammatory disease.

Comments on revised version:

The authors have adequately addressed my concerns.

# Referee report: Reviewer #2 (Public review)

**DOI:** 10.7554/eLife.106338.3.sa2

Uphoff and colleagues present the results of a study focused on characterizing the binding of SVEP1 to TIE1 along with Angiopoietin-2. Starting with computational prediction of SVEP1 binding to TIE1, the authors identify the region of SVEP1 that serves as a high-affinity ligand for TIE1. Advanced studies identify a weak secondary binding site within SVEP1 that appears to be sufficient but not necessary for its interaction with TIE1 based on in vivo rescue experiments. The most novel contribution of the manuscript seems to be the identification of angiopoietin-1 and -2 as co-factors that seem to enhance the binding of SVEP1 with TIE1 and impact downstream AKT signaling. They propose a complex in which SVEP1 binds to TIE1 and ANG2.

Although the first set of results is essentially confirmatory, the identification of ANG-2 as a "co-factor" enhancing the binding of SVEP1 to TIE1 and associated downstream signaling (i.e., Figures 3 and 4) is novel and is of interest. However, the manuscript and its conclusions would greatly benefit from some clarifying details and additional experiments to ensure rigor and support specific claims.

Comments on revised version:

I have no further comments. The authors have addressed my concerns.

# Author comment: Author response

**DOI:** 10.7554/eLife.106338.3.sa3

**Authors:** Katharina Uphoff, Melina Hußmann, Oliver Peters, Matthias Mörgelin, Fabian Metzen, Wolfgang MJ Obermann, Fernando J Bazan, Jörg Stetefeld, Manuel Koch, Stefan Schulte-Merker, Dörte Schulte-Ostermann

**Affiliations:** University of Münster Münster Germany; University of Münster, Present adress: German Cancer Research Center Heidelberg Münster, Heidelberg Germany; University of Manitoba Winnipeg Canada; Colzyx AB Lund Sweden; Univeristy of Cologne Cologne Germany; Univeristy of Cologne Cologne Germany; ħ bioconsulting llc Stillwater United States; University of Manitoba Winnipeg Canada; Univeristy of Cologne Cologne Germany; WWU Muenster Muenster Germany; University of Münster Münster Germany

The following is the authors’ response to the original reviews.

**Public Reviews:**

**Reviewer #1 (Public review):**

Summary:

In this manuscript, Uphoff et al. propose a structural and mechanistic model in which the multidomain ECM protein SVEP1 enables Angiopoietin (ANG) binding to the orphan receptor TIE1, thereby promoting downstream receptor phosphorylation and signaling. Using AlphaFold-based modeling, the authors predict that the CCP20 domain of SVEP1 binds to TIE1, creating a composite surface that facilitates Angiopoietin association and TIE1 activation. The resulting ternary model (SVEP1-TIE1-ANG) offers a structural rationale for how SVEP1 converts TIE1 into a functional, ligand responsive receptor. Additional models and biological assays suggest roles for other domains of SVEP1, such as CCP5-EGF-L7, although these interactions are predicted with low confidence. The authors interpret these findings as the first structural framework for how SVEP1 enables ANG-TIE1 signaling.

Strengths:

(1) The central hypothesis - that SVEP1 enables ANG binding to the orphan receptor TIE1 - is biologically compelling and addresses an important question in vascular biology.

(2) The AlphaFold-predicted ternary complex (SVEP1-TIE1-ANG) is plausible, high-confidence, and structurally consistent with prior functional data (e.g., poly-Ala scanning from Sato-Nishiuchi et al.).

(3) The authors' model offers a potential explanation for the previously observed role of SVEP1 in enhancing ANG signaling through TIE1, and may represent the first structural insight into TIE1's transition from orphan to ligand-activated receptor.

(4) The potential clinical implication - that a combinatorial ligand (ANG+SVEP1) can activate TIE1- could have translational relevance for vascular leak and inflammatory disease.

Weaknesses:

(1) Lack of structural validation and mechanistic follow-up: Despite the promising AlphaFold model, there are no figures of the predicted interface, no residue-level interactions shown, no ipTM values reported, and no experimental follow-up to test the interface. PAE plots are incorrectly used as confidence justifications, which is not appropriate for complex predictions.

We have appended the data showing AlphaFold-predicted interfaces, including residues, hydrogen bonds, and surface complementarity. We also added ipTM scores and confidence plots for the predicted complexes.

(2) Biophysical validation is missing: No surface plasmon resonance (SPR), ITC, or biochemical assays are included to confirm ternary complex formation or quantify binding kinetics. Given the manuscript's structural focus, this is a major gap. For instance, an SPR experiment where ANG is immobilized, and TIE1 binding is measured {plus minus} SVEP1, would directly test the model. And allow direct comparison to ANG-TIE2.

We have addressed this question and performed ELISA assays to measure binding affinities between SVEP1 and TIE1 in presence or absence of ANG1 or ANG2, thus confirming that the affinity is increased in the presence of ANG1 or ANG2.

(3) Missed opportunity for mutagenesis-driven validation: The manuscript does not include any interface-targeted mutations, despite clear opportunities. For example, mutating T2595 in SVEP1 (to R) or mutating the TIE1-specific residues (residues PL 202-203 to LF) could strongly test the model and potentially reveal dominant-negative behaviors. E.g. A T2595 mutant should block ANG binding but not TIE1 binding.

We have depicted figures of the interfaces including P202-L203 and included the TIE1 P202L L203F mutant, as well as the previously described SVEP1 (E2568A - G2569A) mutant in our experimental data. The T2595 mutant was not included in the current study, for the following reason: Modeling suggested that replacing T2595 with an Arg will cause steric and charge clashing with 469GKL471 of ANG1 and 467NKFN470 of ANG2, thus reducing its binding to ANG2 although T2595 does not interact with ANG1/2. A SVEP1 protein comprising CCP15 to the C-terminus with the T2594R mutation shows reduced binding to ANG2, but also reduced binding to TIE1. As the mutation hinders interaction with both TIE1 and ANG2, the data is not included in the manuscript.

(4) Overinterpretation of weak models: The additional AlphaFold model involving the CCP5-EGFL7 domains binding TIE1 has extremely low confidence (ipTM < 0.15) when reexamined by this reader and should not be emphasized. There is no biophysical evidence or binding data (SPR) to support this interaction, and its inclusion detracts from the much stronger CCP20 model.

We agree with this point made by both reviewers and have removed the data on CCP5-EGFL7 from the manuscript.

(5) Language around modeling is overstated and potentially misleading: Terms like "unequivocal," "high-affinity," or "affirms strong binding" in reference to AlphaFold predictions are inappropriate. These are hypotheses -not confirmations - and must be tested at the biochemical level. This should be clarified throughout the manuscript to ensure non-experts do not misinterpret modeling confidence as binding affinity.

We agree with the reviewer, and have adjusted the wording.

(6) Negative stain EM data is not informative due to low resolution and lack of defined interfaces; unless replaced by higher-resolution Cryo-EM, this should be omitted. Better would be co-gel filtration, AUC, or SEC-MALLs with ANG-SVEP1-TIE1.

We have now appended the data by adding gold-labelled TIE/ANG proteins, thus enhancing clarity.

(7) Disjointed narrative: The manuscript presents a compelling mechanism involving CCP20-driven ANG binding to TIE1, but then becomes fragmented by introducing the low-confidence CCP5-EGFL7 model and speculative higher-order polymerization models that are not experimentally supported.

We agree with this point and have have centered the manuscript around CCP20. We removed data concerning CCP5-EGFL7 as suggested by both reviewers.

**Reviewer #2 (Public review):**

Uphoff and colleagues present the results of a study focused on characterizing the binding of SVEP1 to TIE1 along with Angiopoietin-2. Starting with computational prediction of SVEP1 binding to TIE1, the authors identify the region of SVEP1 that serves as a high-affinity ligand for TIE1. Advanced studies identify a weak secondary binding site within SVEP1 that appears to be sufficient but not necessary for its interaction with TIE1 based on in vivo rescue experiments. The most novel contribution of the manuscript seems to be the identification of angiopoietin-1 and -2 as co-factors that seem to enhance the binding of SVEP1 with TIE1 and impact downstream AKT signaling. They propose a complex in which SVEP1 binds to TIE1 and ANG2.

Although the first set of results is essentially confirmatory, the identification of ANG-2 as a "cofactor" enhancing the binding of SVEP1 to TIE1 and associated downstream signaling (i.e., Figures 3 and 4) is novel and is of interest. However, the manuscript and its conclusions would greatly benefit from some clarifying details and additional experiments to ensure rigor and support specific claims.

We have addressed the reviewers concerns and significantly appended the manuscript. Most importantly, we provide structural validation of AlphaFold models reporting interfaces, residue-level interactions and ipTM values. We have included new biophysical validation of binding kinetics of SVEP1 and TIE1 in the presence or absence of ANG1 or ANG2. Furthermore, we have removed the data on CCP5-EGFL7 from the manuscript in order to retain focus on the CCP20 domain.

**Recommendations for the authors:**

**Reviewer #1 (Recommendations for the authors):**

The AlphaFold modeling for the CCP20-based interactions is strong (as determined by this reader rerunning and getting ipTM values and visually inspecting the interactions “because this is not in the manuscript”). As presented, the manuscript stops at the hypothesis-generation stage. Validation is needed to fulfill the paper's title and claims. The structure-function link is not demonstrated, despite an obvious and achievable experimental path (mutagenesis, SPR, kinetics).

Additional Context and Suggestions

(1) Show and label AlphaFold-predicted interfaces, including residues, hydrogen bonds, and surface complementarity.

We have appended the data in the new supplementary figures 1.1, 1.2, 2.1 and 2.2

(2) Provide ipTM scores and confidence plots for each predicted complex.

We have added the values and plots in the new supplementary figures.

(3) Perform SPR assays with ANG-coated surfaces and measure binding of TIE1 {plus minus} SVEP1. Compare to TIE2 binding for context.

We performed the proposed experiment using an ELISA assay to measure binding affinities between SVEP-1 and TIE1 in presence or absence of ANG2 and included these data in the manuscript in figure 2.

(4) Test interface mutants: e.g., T2595R in SVEP1 (should impair ANG recruitment but not TIE1 binding), or PL→LF muta on in TIE1 (should disrupt SVEP1 binding).

We have depicted figures of the interfaces including P202-L203 and included the TIEP202L L203F mutant, as well as the previously described SVEP1 (E2568A - G2569A) mutant in our experimental data. The T2595 mutant was not included in the current study. Our modeling suggested that replacing T2595 with an Arg will cause steric and charge clashing with 469GKL471 of ANG1 and 467NKFN470 of ANG2 thus reduce its binding to ANG2 although T2595 does not interact with ANG1/2. A SVEP1 protein comprising CCP15 to the C-terminus with the T2594R mutation shows reduced binding to ANG2, but also reduced binding to TIE1. As the mutation hinders interaction with both, TIE1 and ANG2, the data is not included in the manuscript.

(5) Clarify in the Introduction that SVEP1 is a large, multidomain ECM protein to help readers contextualize the domain names early on. Do not use terms like CCP before defining them.

We added: “Svep1 encodes a 3571 amino acid long extracellular matrix protein containing different domains such as Willebrand factor type A domain (vWF), ephrin-receptor like domains, complex control protein (CCP) domains, and Hyalin repeats at the N-terminus. The C-terminus mainly consists of CCP and EGF domains. Svep1 is expressed in mesenchymal cells, but not in endothelial cells, and functions non-cell-autonomously (Karpanen et al. 2017; Morooka et al. 2017)”.

(6) Replace or remove negative-stain EM unless higher-resolution cryo-EM data are available.

We would like to retain the EM data, but have now replaced the negative-stain EM by adding gold-labelled TIE/ANG Proteins to verify that the proteins we show are the ones we expect. The reason we would like to retain the data is that TIE1 has been considered for so many years as an orphan receptor, and thus we consider it appropriate to demonstrate SVEP1/TIE1 binding using multiple methods.

(7) Reframe claims around modeling to avoid overstatement. For example: "The model suggests a plausible mechanism consistent with prior biochemical data" is more appropriate than "unequivocal".

We agree with the reviewer, and have adjusted the wording.

(8) Consider narrowing the focus: the CCP20-TIE1-ANG model is a strong story on its own. The CCP5EGFL7 model and polymerization hypotheses are not essential and may dilute the impact.

Since this point was made by more than one reviewer, we have removed the data on CCP5EGFL7 from the manuscript.

(9) Properly define TIE1: Tyrosine kinase with Ig and EGF domains.

We have corrected the full protein name for TIE1 and added: “TIE1 and Tie2 exhibit a high degree of homology with a globular head domain consisting of three immunoglobulin-like (Ig) domains and three epidermal growth factor-like (EGF) modules and a short stalk formed by three fibronectin type III repeats, while the N-terminal two Ig domains of Tie2 harbor the angiopoietin binding site (Macdonald et al. 2006).” We also added: “D1 and D2 refer to the two N-terminal Ig domains, D3 refers to the three EGF domains and D4 to the third Ig domain of TIE1 or TIE2.”

(10) Use RTK, not tyrosine kinase receptors TKR.

Tyrosine Kinase receptor was replaced by receptor tyrosine kinases (RTKs)

(11) PDBs (.cif and .json files) of the models must be supplied for the readers so they don't need to rerun the AlphaFold jobs.

We are providing all PDBs with the revised manuscript.

**Reviewer #2 (Recommendations for the authors):**

Major comments:

(1) In several locations, the authors state that alphafold detects an "unequivocal" and/or "high affinity" interaction. Experts on computational structure prediction can weigh in, but I am not sure it is accurate to say that alphafold predicts affinity. Quantitative estimates of the prediction confidence or other parameters of the alphafold output are not provided.

Thank you for the comment, we agree with the reviewer and have adjusted the wording. We have also appended the data showing interfaces, ipTM scores and confidence plots for the predicted complexes.

(2) In Figure 1C, what concentrations of TIE1 and TIE2 are being used in the SPR experiments shown?

We have added the concentrations of the proteins to the methods section

(3) In Figure 1C, what is the affinity constant (KD) of the interaction between SVEP1 and TIE1 and SVEP1 and TIE2?

We have added the values of affinity constants to the manuscript text.

(4) In Figure 1C, the authors immobilize a "70kD" fragment of human SVEP1 to determine the interaction between SVEP1 and TIE1. How does the affinity they measure between the SVEP1 fragment and TIE1 compare to the affinity between immobilized full-length SVEP1 with TIE1?

The largest molecule we used in any assay is not full-length SVEP1, but consisted of a C-terminal SVEP1 protein spanning from the first EGF domain to the C-terminus (approximately 295 kDa) as previous studies have shown that SVEP1 is proteolytically cleaved N-terminal to the first EGF domain, generating a protein of this size. In our ELISA assays, this molecule has a higher affinity to TIE1 than the 70 kD fragment. We have included data for the 70 kDa as well as for the 295 kDa fragment in the manuscript. ELISA assays have used larger SVEP1 fragments (as indicated in the figure legends), the SPR assay was performed with the 70 kDa fragment.

(5) How does the alphafold structure prediction for the "70kD" fragment of hSVEP1 compare to the prediction of the same 70kD fragment from the full-length protein prediction?

All modellings using different SVEP1 fragments including the 70 kD and full-length version identify the putative binding site at position CCP20. While AlphaFold3 can predict the correct domain folds in both the 70kDa fragment and full-length SVEP1, the orientation of these domains is highly variable due to flexible linkers between each domain module. This flexibility effects the output confidence metrics, thereby hampering our interpretation of the models. Therefore, we conducted the structural prediction of the complexes with smaller fragments and not the full-length SVEP1.

(6) What are the amino acids for the 70kD fragment?

The relevant amino acids are 2261-2890. The accession number and amino acids of each protein have been listed in the key resources table.

(7) In Figure 1D, what is being depicted by the red stars? This is not explained in the text or figure legend.

We have replaced figure 1D.

(8) By itself, Figure 1D is not terribly informative and in my opinion does not support the statement that the authors "were able to directly visulalize the attachment of SVEP1 and TIE1." As a minimum, the authors should repeat the same set of images with SVEP1 and TIE2, but other approaches, such as labeling, could be performed.

We replaced figure 1D with new data and gold-coated protein enhancing clarity. We think it beneficial to demonstrate SVEP1/TIE1 binding using multiple methods as TIE1 has been considered as an orphan receptor for so many years. We have not performed these experiments with TIE2 proteins as we were not able to show binding of TIE2 to SVEP1 with other assays.

(9) What is being stained in Figure 1D? Full-length SVEP1/TIE1? Or fragments of these proteins?

We replaced figure 1D by a new assay with labeled proteins using the 150 kDa version of SVEP1 and the ectodomain of TIE1 as well as ANG1 or ANG2 (new figure 1D and new supplementary figure 2.3). TIE1/ANG proteins were gold-labelled. The protein fragments used in this assay are described in detail in the methods section.

(10) The authors discover CCP6-EGFL7 as a low-affinity binding region of SVEP1 for TIE1. Is this region in physical proximity to CCP20 (the high-affinity binding region for TIE1) based on alphafold prediction? How would the authors think this is binding TIE1?

We have removed this data set (see comment to reviewer 1’s request).

(11) What is the affinity constant (KD) for CCP6-EGFL7 with TIE1?

We have removed this data set (see comment to reviewer’s 1 request).

(12) The authors claim that ANG1/ANG2 increase affinity between SVEP1 and TIE1 based on immunoblotting. Immunoblots are semi-quantitative at best. If the claim is higher affinity, I think the authors should measure this by SPR and determine the KD between immobilized SVEP1 with TIE1 in the absence and presence of ANG1 and/or ANG2.

We conducted ELISA assays (figure 2) showing that the affinity is increased in the presence of ANG1 or ANG2 and agree with this reviewer that this strengthens the data.

(13) In Figure 3, can the authors explain why ANG1/2 does not pull down with SVEP1/TIE1?

We noticed that upon transfection of TIE1 into HEK cells, ANG1/2 is almost undetectable anymore in the total lysate. Thus, we believe that after the pull down we are below the detection limit.

(14) In Figure 3, what is "TL"? I assume total lysate, but this is not specified.

Thank you, we now specify TL as total lysate.

(15) In Figure 3 "TL" panel (again, I assume this is total lysate), why are the ANG1/2 immunoblots so weak when co-transfected with TIE1?

We consider it likely that in the presence of TIE1, ANG1/2 proteins are internalized and digested. Another reason for low signals could be that upon transfection of two plasmids, the amount of ANG1/2 protein is reduced as the cell has limited capacity for transcription and translation.

(16) In Figure 3B, why is the SVEP1 fragment now 150kD when 70kD fragment was previously used? What domains are contained in this 150kD fragment?

We now better define the domains of the 150kD SVEP1 fragment. The 150 kD fragment was the one produced first and available in high amounts in our laboratory and thus used for functional assays. The 70 kDa fragment together with ANG2 also induces phosphorylation of AKT, but it was not used in as many conditions/replicates as the amounts we had available were lower.

(17) In Figure 4A, signaling with SVEP1 by itself and ANG2 by itself should be shown to support the claims being made.

We added the lines for SVEP1 and ANG2, and also the quantification. SVEP1 itself already affects the phosphorylation of TIE1, most likely because hdLECs produce ANG2 by themselves. We show this with the ANG2 blocking antibody for pAKT.

(18) For pAKT, what are the concentations of proteins being used and the times of incubation?

This information is provided in the Materials and Methods section. We added the concentration of the anti-ANG2 antibody, which was missing.

(19) It seems that p-AKT and AKT are being blotted on different gels. If this is correct, loading controls need to be shown for p-AKT blot.

We added HSC70 as a loading control for both blots.

(20) It is interesting that anti-ANG2 antibody inhibits SVEP1-induced p-AKT signaling. As the authors may know, SVEP1 has been identified as a receptor for PEAR1, which also leads to downstream p-AKT signaling, which seems to be independent of ANG2. Do the LECs being used here express PEAR1? If these cells express PEAR1, how do the authors think ANG2 silencing will eliminate SVEP1-associated p-AKT signaling?

hdLECs express PEAR1. However, we show that p-AKT signaling is attenuated after siRNA KO of TIE1. Thus, the downstream signaling is dependent on TIE1 (Figure3).

(21) Again, experts on computational structure prediction can weigh in, but I am not sure how to interpret the prediction of the 2:2:2 stoichiometry for the theoretical SVEP1/TIE1/ANG1-2 complex. Are there quantitative estimates of the confidence that can be provided? Did the authors attempt to model this complex with different stoichiometries? It is difficult to know how relevant this model is without any experimental results supporting this result.

Since 1:1:1 is the smallest possible triple complex, it is our starting point. We can model a 2:2:2 version, but anything larger than this AlphaFold will not run. Furthermore, we now provide quantitative estimates of confidence with the pLDDT, PAE, pTM, ipTM scores for all models including the 2:2:2 complexes.

Minor comments:

(1) The authors could consider including a reference to alphafold on line 102.

We have added a reference for AlphaFold2 and 3

(2) The authors should refer to surface plasmon resonance (SPR) assays by this term as opposed to using the brand name Biacore.

We agree with the reviewer and have changed the term Biacore to SPR.
